# Geographical variation of overweight, obesity and related risk factors: Findings from the European Health Examination Survey in Luxembourg, 2013-2015

**DOI:** 10.1371/journal.pone.0197021

**Published:** 2018-06-14

**Authors:** Hanen Samouda, Maria Ruiz-Castell, Valery Bocquet, Andrea Kuemmerle, Anna Chioti, Frédéric Dadoun, Ngianga-Bakwin Kandala, Saverio Stranges

**Affiliations:** 1 Epidemiology and Public Health Research Unit, Population Health Department, Luxembourg Institute of Health, Strassen, Luxembourg; 2 Competence Center for Methodology and Statistics, Luxembourg Institute of Health, Strassen, Luxembourg; 3 Swiss Tropical and Public Health Institute, Basel, Switzerland; 4 Endocrinology and Diabetology Department, Centre Hospitalier de Luxembourg, Luxembourg, Luxembourg; 5 Department of Mathematics, Physics and Electrical Engineering, Faculty of Engineering and Environment, Northumbria University, Newcastle upon Tyne, United Kingdom; 6 Department of Epidemiology and Biostatistics, Schulich School of Medicine & Dentistry, Western University, London, ON, Canada; CUNY, UNITED STATES

## Abstract

The analyses of geographic variations in the prevalence of major chronic conditions, such as overweight and obesity, are an important public health tool to identify “hot spots” and inform allocation of funding for policy and health promotion campaigns, yet rarely performed. Here we aimed at exploring, for the first time in Luxembourg, potential geographic patterns in overweight/obesity prevalence in the country, adjusted for several demographic, socioeconomic, behavioural and health status characteristics. Data came from 720 men and 764 women, 25–64 years old, who participated in the European Health Examination Survey in Luxembourg (2013–2015). To investigate the geographical variation, geo-additive semi-parametric mixed model and Bayesian modelisations based on Markov Chain Monte Carlo techniques for inference were performed. Large disparities in the prevalence of overweight and obesity were found between municipalities, with the highest rates of obesity found in 3 municipalities located in the South-West of the country. Bayesian approach also underlined a nonlinear effect of age on overweight and obesity in both genders (significant in men) and highlighted the following risk factors: 1. country of birth for overweight in men born in a non-European country (Posterior Odds Ratio (POR): 3.24 [1.61–8.69]) and women born in Portugal (POR: 2.44 [1.25–4.43]), 2. low educational level (secondary or below) for overweight (POR: 1.66 (1.06–2.72)] and obesity (POR:2.09 [1.05–3.65]) in men, 3. single marital status for obesity in women (POR: 2.20 [1.24–3.91]), 4.fair (men: POR: 3.19 [1.58–6.79], women: POR: 2.24 [1.33–3.73]) to very bad health perception (men: POR: 15.01 [2.16–98.09]) for obesity, 5. sleeping more than 6 hours for obesity in unemployed men (POR: 3.66 [2.02–8.03]). Protective factors highlighted were: 1. single marital status against overweight (POR: [0.60 (0.38–0.96)]) and obesity (POR: 0.39 [0.16–0.84]) in men, 2. the fact to be widowed against overweight in women (POR: [0.30 (0.07–0.86)], as well as a non European country of birth (POR: 0.49 [0.19–0.98]), tertiary level of education (POR: 0.34 [0.18–0.64]), moderate alcohol consumption (POR: 0.54 [0.36–0.90]) and aerobic physical activity practice (POR: 0.44 [0.27–0.77]) against obesity in women. A double burden of environmental exposure due to historic mining and industrial activities and past economic vulnaribility in the South-West of the country may have participated to the higher prevalence of obesity found in this region. Other demographic, socioeconomic, behavioural and health status covariates could have been involved as well.

## Introduction

Overweight and obesity are regarded as a public health priority, both because of their pandemic progression with more than 2 billions people impacted worldwide, namely about 28% of the world population, and owing to the potentially relatedburden of diseases [1–3].

The latest systematic analysis performed in the frame of the the Global Burden of Disease Study estimated the prevalence of obesity around 23.7% for men and 26% for women in Luxembourg. Overweight prevalence estimation was about 34.3% in men and 18.4% in women (data collected before 2013) [1].

Obesity is a complex disease, with a multiple etiology, including foetal programmation, genetic predisposition, physiological mechanisms, psychological factors, food production and behavior, physical activity pattern, environmental and social context [4–6].

Demographic, behavioural and socioeconomic risk factors such as increased age, lower education level and employment, unbalanced dietary habits and/or physical inactivity have previously been highlighted as correlates of obesity in Luxembourg and worldwide [7–11].

Overweight and obesity have also been linked to environmental risk factors such as the urban area of residence, in spite of existing controversies especially between urban and rural areas [12–16]. A north-south decreasing gradient of obesity has also been underlined in several countries [17]. In Luxembourg, Tchikaya *et al*. found a higher likelihood of obesity amongst people living outside the capital, in particular in the North and the West areas of the country [11]. Westernisation has also been suggested as an additional driver of the obesity increase in several countries [18, 19]. Recent evidence has also suggested the influence of factors such as environmental chemicals on body weight disorders [20]. Nevertheless, individual intrinsic and environmental extrinsic risk factors tend to be separately considered in the literature, in spite of their close interconnection [7–13, 17–19].

Geographic variation analyses address the challenge to combine prevalence of overweight and obesity with both individual and environmental risk factors through an integrative spatial approach. Geographic variation analyses are mainly based on Bayesian statistical approaches and, therefore, also investigate both observed and random data [21–23]. Through the geographic variation analyses based on Bayesian approaches, certain authors called into question some concepts previously underlined by the logistic statistical approaches usually used, such as the north-south decreasing gradient of obesity [17, 21]. Analyses of geographic variations in the prevalence of major chronic conditions, such as overweight/obesity, are an important public health tool to identify “hot spots”, which would be of a very high concern especially in Luxembourg. Indeed, in spite of its small surface area of approximately 2 586 km^2^, Luxembourg shows a complex territory organization with no less than 106 administrative municipalities, which, despite their organization in 3 main districts (Diekirch, Grevenmacher and Luxembourg), remain differently influenced by the geographic location. Several municipalities could indeed be influenced by more than one neighbouring country. Some municipalities located in the district of Diekirch could be influenced by both Belgian (North-West side) and German (North-East side) genetic predisposition, environment and behaviours. Others located in the disctrict of Luxembourg could be impacted by both Belgian and French neighbourhood, respectively situated on the West- and South-side. Municipalities located in Grevenmacher district could be influenced by German (Centre-East side) and/or French specificities (South-Est side).

**The aim** of the present paper was to investigate the geographical variation of overweight and obesity risk in Luxembourg, adjusted for several demographic, socioeconomic, behavioural and health status characteristics.

We used the most recent population-based data, from the European Health Examination Survey, conducted in Luxembourg between 2013 and 2015 [24, 25].

## Materials and methods

### European Health Examination Survey in Luxembourg (EHES-LUX_2013–2015_)

Data came from EHES-LUX_2013-2015_ conducted amongst the 25–64 years old noninstitutionalized resident population of Luxembourg, from February 2013 to January 2015 [24–26]. 1529 participants were invited to take part to the survey following a random selection (One-stage sampling procedure from the national population register managed by the “Inspection générale de la sécurité sociale” in Luxembourg, with age-, gender- and district of residence stratification) [26, 27].

For the purpose of this analysis, data of 24 participants having a BMI <18.5 kg/m^2^ and 21 pregnant women were excluded [28]. We analysed the data of 1484 subjects, men (N = 720) and women (N = 764). Participants gave written informed consent (S1 File: Consent Form). National Ethics of Research and Data Protection Committees authorized the study.

The European Health Examination Survey (S2 File: EHES Questionnaire) includes: a questionnaire (on demographic, socioeconomic, behavioural, geographic and health status items) and a clinical examination (including weight and height measurement).

### Overweight and obesity definition

In the clinical examination part, weight and height were measured by trained nurses, based on a standardised protocol developed in the framework of EHES-LUX [25].

Body Mass Index (BMI) was calculated as individual’s weight divided by the square of the height (kg/m^2^). Overweight (BMI of 25–29.9 kg/m^2^) and obesity (BMI ≥ 30 kg/m^2^) prevalence were calculated, based on the population of the 25–64 years-old residents in Luxembourg in 2014 [29].

### Demographic, socioeconomic and geographic determinants

We analysed demographic, socioeconomic, behavioural, geographic and health status data from Ehes-Lux_2013-2015_: age and gender, administrative municipality of residence, administrative district area of residence (North: Diekirch. Centre: Luxembourg. East: Grevenmacher), country of birth (according to the political division, Luxembourg and Portugal being the two most prevalent birth places for Luxembourg residents), marital status (single: “never married nor in civil partnership”, “married and/or in partnership”, “divorced and/or in dissolved civil partnership”, “widowed and/or surviving to partner death”), employment status (“employed” carrying out a job or profession and “unemployed”) and the household total net monthly income [24–26].

### Behavioural aspects

Data related to the daily consumption of fruits and vegetables were analysed (number of portions and frequency of consumption: “once or more a day” or “less than once a day” [25]). We assessed physical activity, based on 4 indicators, according to EHIS-PAQ questionnaire: 1.WRPA (work-related physical activity): “percentage of people mostly physically active when working” [30]; 2.TRPA (transport-related physical activity): “metabolic equivalent per minute (MET/minute) and per week of walking and cycling to reach the workplace, expressed in quintiles” [30]; 3.HEPA (Health-enhancing physical activity), which includes: -APA (Aerobic PA guideline compliance): “percentage of people performing at least 150 min aerobic physical activity per week” [30] and -MSPA (muscle strengthening physical activity): “percentage of people practicing a muscle strengthening physical activity at least 2 days per week” [30].

To evaluate the weekly standard alcoholic drink consumption, we developed a 3-category indicator based on the product of “the weekly frequency of alcoholic drink” by “the number of standard drinks per day defined as 30 centilitres (cl) of beer, 15cl of champagne, 15cl of wine, 4cl of spirits, 8cl of aperitif cocktails, 4cl of shots and/or 1 tall glass of spirits or soft drink”:—Category 1: ‘No drinker or seldom’ (< once a week consumption).—Category 2: ‘Low drinker’ (1 to 6 drinks a week).—Category 3: ‘High drinker’ (> 6 drinks a week). As both variables “weekly frequency” and “daily number of standard drinks” contained ranges as choices, the median values were used, 6 drinks being the median value of the alcoholic weekly consumption [24, 25].

### Self-perceived health, physical and mental health condition

Self-perceived health was evaluated through the question “how is your health in general?”, with answers separated in 3 categories: “Very good to good”, “fair” and/or “bad to very bad” health. People were also asked to document “the intensity of physical pain they had suffered during the past 4 weeks”: “from low intensity to no pain”, “moderate pain” or “severe or very severe pain” [25]. Employed participants were enquired about their “usual sleep duration per night for nights preceding a work day” [24, 25]. For unemployed participants, the following question was asked: “how many hours do you sleep normally at night when you do not work the next day?”. We created a 4-categories indicator gathering sleep duration and labour status:—Employed participants with sleep duration ≤ 6h;—Employed participants with sleep duration > 6h;—Unemployed participants with sleep duration ≤ 6h;—Unemployed participants with sleep duration > 6h. Presence of symptoms of depression was defined by PHQ-9 (*Patient Health Questionnaire*) score ≥ 5, based on the daily frequency to have “little interest to do things”, “depressive or hopeless feelings”, “hypersomnia or insomnia”, “tiredness”, “eating disorders”, “failure feeling”, “concentration problems”, “slow speaking and moving or fidgeting” and/or “dead or self-harming ideas” [25, 31].

The one hundred six municipalities of residence were used to investigate geographic distribution of overweight and obesity.

### Statistical analyses

Descriptive analyses were expressed in percentages, tertiles, means, standard deviations, minimum and/or maximum values. Chi-square (χ^2^) and Mann–Whitney U-tests were performed to assess the statistical significance of overweight/obesity prevalence and risk factors. To estimate the likelihood of overweight or obesity, we used logistic regression analyses where continuous variables were centred. Selection of variables was based on a literature review and on statistical criteria (variables showing p< 0.20 in the univariate logistic analyses were entered in the multivariable logistic models). Odds Ratio (OR) were assessed. Multicollinearity between explanatory variables for BMI was checked. Interactions were verified. To account for the stratified random sampling method used to recruit the participants, weighted statistical methods were applied to produce nationally representative estimates. A sampling weight equal to the inverse probability of unit selection was allocated to each participant from the same stratum. This stratum sampling weight was defined as the ratio between the population and the observed sample stratum sizes (SAS version 9.4, SAS Institute Inc., Cary, NC, USA).

Moreover, we investigated the geographical variation of overweight/obesity prevalence, adjusted for demographic, socioeconomic, behavioural and health status characteristics at the municipality level and in comparison with normal weight. To take into account the potential nonlinear effects of overweight/obesity risk factors, we used a geo-additive semi-parametric mixed model and a Bayesian approach based on the Markov Chain Monte Carlo (MCMC) techniques for inference and model checking [21, 26, 27, 32]. This approach investigate both observed and random data [21, 23]. The response variable was defined as *y*_*i*_ *= 1 if overweight or obesity and y*_*i*_ *= 0 for normal weight*. The standard measure of effect was the posterior odds ratio (POR) and 95% credible region [21, 26, 32]. Multivariate Bayesian geo-additive modelisation was performed in order to assess POR significance calculated for the fixed, nonlinear and spatial effects. Results were considered to be significant at the 5% critical level (CR) (p<0.05). Bayesian analyses took into account the effect of the neighbouring municipalities (also called communes in Luxembourg) in the models developed. POR were represented on the map of Luxembourg, for each municipality. Red coloured areas represented the municipalities at high risk of overweight/obesity, while green coloured areas illustrated those at low risk. The significance of the spatial effect was reported on a second map: black coloured areas illustrated significant positive spatial effect at the municipalities level. White coloured areas represented significant negative spatial effect and grey coloured areas concerned no significant effect. Bayes X software package, version 2.0.1, was used in order to perform the Bayesian analyses.

## Results

### Prevalence of overweight and obesity between 2013 and 2015 in Luxembourg

High rates of overweight (37.3%) and obesity (20.6%) were observed in Ehes-Lux_2013-2015_, which, once extended to the general adult population residing in Luxembourg between 2013 and 2015 [29], would represent a prevalence of overweight of approximately 38%, and a prevalence of obesity of about 20,2% (Table 1).

**Table 1:** Prevalence of normal weight, overweight and obesity in Luxembourg. According to EHES-LUX_2013-2015_ Survey.

	Luxembourgisch Adult Population (25–64 years-old) (N = 313.586 in 2014)[29]					
Prevalence	General Population		Men		Women	
	Prevalence%	95% CI ^a^	Prevalence%	95% CI ^a^	Prevalence%	95% CI ^a^
Normal weight	41.83%	39.27–44.38	32.18%	28.67–35.69	52.31%	48.73–55.90
Overweight	37.96%	35.43–40.49	46.77%	43.04–50.51	28.38%	25.15–31.62
Obesity	20.21%	18.15–22.27	21.05%	18.04–24.05	19.30%	16.49–22.11

^**a**^ 95% CI: 95% confidence interval.

General characteristics are detailed in Table 2.

**Table 2:** Normal weight, overweight and obesity in EHES-LUX_2013-2015_ Survey: General characteristics.

	EHES-LUX2013-2015—N = 148			Women—N = 764				Men—N = 720			
	Women N = 764	Men N = 720	P-value	Normal Weight N = 396 (51.8%)	Overweight N = 218 (28.5%)	Obesity N = 150 (19.6%)	P-value	Normal Weight N = 227 (31.5%)	Overweight N = 337 (46.8%)	Obesity N = 156 (21.7%)	P-value
**I. ANTHROPOMETRY**											
**Body Mass Index (Kg/m2)**			< 0.0001				<0.0001				<0.0001
Mean ± sd	26.1 ± 5.4	27.4 ± 4.4		22.2 ± 1.7	27.1 ± 1.4	34.8 ± 4.4		23.1 ± 1.4	27.4 ± 1.4	33.8 ± 3.6	
(min-max)	(18.6–54.2)	(18.7–52.6)		(18.6–25)	(25.0–30.0)	(30.0–54.2)		(18.7–25.0)	(25.0–30.0)	(30.0–52.6)	
**II. DEMOGRAPHIC, SOCIOECONOMIC AND GEOGRAPHIC CHARACTERISTICS**											
**Age (y)**			0.95				0.0001				0.0001
Mean ± sd	45.1 ± 10.3	45.1 ± 9.9		43.6 ± 10.2	46.5 ± 10.4	47.1 ± 9.7		43.1 ± 9.8	45.1 ± 9.7	47.9 ± 9.6	
(min-max)	(25.7–65.0)	(26.2–65.0)		(26.3–65.0)	(25.7–65.0)	(26.3–64.8)		(26.6–64.7)	(26.6–65.0)	(26.3–65.0)	
**District areas of residence (%)**			0.34				0.95				0.45
- Diekirch	113 (100.0%)	89 (100.0%)		57 (50.5%)	32 (28.3%)	24 (21.2%)		24 (27.0%)	47 (52.8%)	18 (20.2%)	
- Grevenmacher	102 (100.0%)	106 (100.0%)		51 (50.0%)	32 (31.4%)	19 (18.6%)		29 (27.4%)	49 (46.2%)	28 (26.4%)	
- Luxembourg	549 (100.0%)	525 (100.0%)		288 (52,4%)	154 (28.1%)	107 (19.5%)		174 (33.1%)	241 (45.9%)	110 (21.0%)	
**Country of birth (%)**			0.58				< 0.0001				0.03
- Luxembourg	387 (100.0%)	388 (100.0%)		203 (52.4%)	111 (28.7%)	73 (18,9%)		132 (34.0%)	162 (41.8%)	94 (24.2%)	
Portugal	113 (100.0%)	106 (100.0%)		34 (30.1%)	42 (37.2%)	37 (32,7%)		30 (28.3%)	53 (50.0%)	23 (21.7%)	
- Other European*	181 (100.0%)	158 (100.0%)		113 (62.4%)	41 (22.7%)	27 (14,9%)		49 (31.0%)	78 (49.4%)	31 (19.6%)	
- No European**	83 (100.0%)	68 (100.0%)		46 (55.4%)	24 (28.9%)	13 (15,7%)		16 (23.5%)	44 (64.7%)	8 (11.8%)	
**Marital status (%)**			< 0.0001				0.33				0.002
- Single	131 (100.0%)	164 (100.0%)		70 (53.4%)	36 (27.5%)	25 (19.1%)		72 (43.9%)	69 (42.1%)	23 (14.0%)	
- Married/In registered partnership	500 (100.0%)	482 (100.0%)		264 (52.8%)	142 (28.4%)	94 (18.8%)		139 (28.8%)	234 (48.6%)	109 (22.6%)	
- Widowed/Surviving partner death	24 (100.0%)	4 (100.0%)		16 (66.7%)	4 (16.7%)	4 (16.7%)		1 (25.0%)	2 (50.0%)	1 (25.0%)	
- Divorced/Dissolved Partnership	109 (100.0%)	70 (100.0%)		46 (42.2%)	36 (33.0%)	27 (24.8%)		15 (21.4%)	32 (45.7%)	23 (32.9%)	
**Educational level (%)**			0.16				< 0.0001				0.01
-Primary / Lower secondary	192 (100.0%)	176 (100.0%)		66 (34.4%)	60 (31.2%)	66 (34.4%)		52 (29.6%)	86 (48.9%)	38 (21.5%)	
-UpperPost-secondary / No-tertiary	309 (100.0%)	264 (100.0%)		157 (50.8%)	95 (30.7%)	57 (18.5%)		68 (25.8%)	127 (48.1%)	69 (26.1%)	
-Tertiary education	260 (100.0%)	278 (100.0%)		172 (66.1%)	62 (23.9%)	26 (10.0%)		107 (38.5%)	122 (43.9%)	49 (17.6%)	
**Employment status (%) (N = 1483)**			< 0.0001				0.01				0.55
- Employed people	548 (100.0%)	592 (100.0%)		296 (54.0%)	159 (29.0%)	93 (17.0%)		190 (32.1%)	278 (47.0%)	124 (20.9%)	
- Unemployed people	216 (100.0%)	127 (100.0%)		100 (46.3%)	59 (27.3%)	57 (26.4%)		37 (29.1%)	58 (45.7%)	32 (25.2%)	
**Income- Quintiles Euros (N = 1344)**			< 0.0001				0.003				0.005
- Quintile 1	3300	3750		3600	3200	3100		4000	3500	3800	
- Quintile 2	4625	5500		5000	4500	4225		6000	5250	5000	
- Quintile 3	6500	7750		7125	6500	5500		8500	7500	7600	
**III. BEHAVIOURAL ASPECTS**											
**Physical Activity** **Work related physical activity (%) N = 1480**		203 (100.0%) 407 (100.0%) 110 (100.0%)	0.0005				0.003				0.09
- Yes	230 (100.0%)			106 (46.1%)	71 (30.9%)	53 (23.0%)		57 (28.1%)	141 (53,2%)	38 (18.7%)	
- No	362 (100.0%)			213 (58.8%)	95 (26.2%)	54 (14.9%)		141 (34.6%)	178 (43.7%)	88 (21.6%)	
- Not working	168 (100.0%			75 (44.6%)	51 (30.4%)	42 (25.0%)		29 (26.4%)	51 (46.4%)	30 (27.3%)	
**Transport-related physical activity Metabolic equivalent/min-** N = 1476			0.36				0.95				0.58
- Quintile 1	132	132		132	132	132		132	66	0	
- Quintile 2	396	445.5		396	396	330		445.5	445.5	330	
- Quintile 3	1039.5	1039.5		1039.5	834	742.5		1039.5	996	891	
**Aerobic physical activity** N = 1480%			0.07				< 0.0001				0.0002
- Yes	269 (100.0%)	287 (100.0%)		165 (61.3%)	72 (26.8%)	32 (11.9%)		110 (38.3%)	135 (47.0%)	42 (14.6%)	
- No	492 (100.0%)	432 (100.0%)		230 (46.7%)	146 (29.7%)	116 (23.6%)		117 (27.1%)	202 (46.8%)	113 (26.2%)	
**Muscle- strengthening** (%) N = 1481			0.52				0.02				0.006
- Yes	153 (100.0%)	154 (100.0%)		93 (60.8%)	40 (26.1%)	20 (13.1%)		57 (37.0%)	78 (50.7%)	19 (12.3%)	
- No	609 (100.0%)	565 (100.0%)		302 (49.6%)	178 (29.2%)	129 (21.2%)		170 (30.1%)	259 (45.8%)	136 (24.1%)	
**Fruits / Vege (N = 142)**			< 0.0001				0.64				0.81
**Consumption of fruit (%)**											
-Once or more a day	459 (100.0%)	330 (100.0%)		244 (53.2%)	129 (28.1%)	86 (18.7%)		106 (32.1%)	156 (47.3%)	68 (20.6%)	
-Less than once a day	303 (100.0%)	390 (100.0%)		151 (49.8%)	89 (29.4%)	63 (20.8%)		121 (31.0%)	181 (46.4%)	88 (22.6%)	
**Consumption of vegetables (%)**			< 0.0001				0.29				0.36
-Once or more a day	500 (100.0%)	321 (100.0%)		267 (53.4%)	143 (28.6%)	90 (18.0%)		105 (32.7%)	141 (43.9%)	75 (23.4%)	
-Less than once a day	262 (100.0%)	399 (100.0%)		128 (48.9%)	75 (28.6%)	59 (22.5%)		122 (30.6%)	196 (49.1%)	81 (20.3%)	
**Number of fruits, vegetables portions per day** (N = 1480)			< 0.0001				0.31				0.64
Mean ± sd	2.70 ± 2.25	1.86 ± 2.11		2.79 ± 2.30	2.65±2.22	2.47± 2.13		1.89 ± 2.12	1.79± 2.04	1.97± 2.25	
(min-max)	(0–12)	(0–12)		(0–12)	(0–11)	(0–9)		(0–12)	(0–9)	(0–12)	
**Alcohol (%) (N = 1481)**			< 0.0001				0.01				0.41
-No drinker	411 (100.0%)	184 (100.0%)		193 (47.0%)	121 (29.4%)	97 (23,6%)		57 (31.0%)	83 (45.1%)	44 (23.9%)	
-1-6 drinks a week	236 (100.0%)	231 (100.0%)		138 (58.5%)	66 (28.0%)	32 (13,5%)		75 (32.5%)	116 (50.2%)	40 (17.3%)	
-More than 6 drinks a week	114 (100.0%)	305 (100.0%)		64 (56.1%)	30 (26.3%)	20 (17.5%)		95 (31.1%)	138 (45.3%)	72 (23.6%)	
**IV. HEALTH DETERMINANTS**											
**Self-perceived health** (N = 1483)			0.15				<0.0001				<0.0001
Very good to good	564 (100.0%)	562 (100.0%)		320 (56,7%)	161 (28.6%)	83 (14.7%)		193 (34.3%)	267 (47.5%)	102 (18.2%)	
Fair	164 (100.0%)	127 (100.0%)		63 (38,4%)	46 (28.1%)	55 (33.5%)		31 (24.4%)	54 (42.5%)	42 (33.1%)	
Bad to very bad	35 (100.0%)	31 (100.0%)		13 (37,1%)	11 (31.4%)	11 (31.4%)		3 (9.7%)	16 (51.6%)	12 (38.7%)	
**Physical pain, discomfort** (%) (N = 1482)			< 0.0001				0.01				0.61
None, very mild to mild	533 (100.0%)	572 (100.0%)		291 (54.6%)	153 (28.7%)	89 (16.7%)		185 (32.3%)	269 (47.0%)	118 (20.6%)	
Moderate	159 (100.0%)	96 (100.0%)		77 (48.4%)	41 (25.8%)	41 (25.8%)		29 (30.2%)	44 (45.8%)	23 (24.0%)	
Severe or very severe	70 (100.0%)	52 (100.0%)		27 (38.6%)	24 (34.3%)	19 (27.1%)		13 (25.0%)	24 (46.2%)	15 (28.8%)	
**Sleep duration** (%) (N = 1476)			< 0.0001				0.06				0.03
Employed, sleep duration < = 6h	128 (100.0%)	207 (100.0%)		63 (49.2%)	36 (28.1%)	29 (22.7%)		53 (25.6%)	97 (46.9%)	57 (27.5%)	
Employed, sleep duration > 6h	417 (100.0%)	383 (100.0%)		230 (55.2%)	123 (29.5%)	64 (15.3%)		137 (35.8%)	180 (47.0%)	66 (17.2%)	
Unemployed, sleep duration< = 6h	57 (100.0%)	34 (100.0%)		25 (43.9%)	18 (31.6%)	14 (24.6%)		7 (20.6%)	19 (55.9%)	8 (23.5%)	
Unemployed, sleep duration > 6h	158 (100.0%)	92 (100.0%)		75 (47.5%)	41 (25.9%)	42 (26.6%)		29 (31.5%)	39 (42.4%)	24 (26.1%)	
**Depression** (%) (N = 1482)			< 0.0001				0.007				0.70
Yes	200 (100.0%)	119 (100.0%)		91 (45.5%)	55 (27.5%)	54 (27.0%)		35 (29.4%)	55 (46.2%)	29 (24.4%)	
No	562 (100.0%)	601 (100.0%)		304 (54.1%)	163 (29.0%)	95 (16.9%)		192 (32.0%)	282 (46.9%)	127 (21.1%)	

### Demographic, socioeconomic, behavioural and health status correlates

Univariate analyses are detailed in S1 and S2 Tables.

#### Overweight

Table 3 in men and Table 4 in women detail the OR (logistic regressions) and POR (Bayesian modelisation) of overweight and obesity according to their respective covariates.

**Table 3:** Multivariable logistic and Bayesian predictive models of overweight and obesity in men, in relation to normal weight (EHES-LUX_2013-2015_).

Men	Overweight (N = 562)		Obesity (N = 378)	
	Logistic	Bayesian	Logistic	Bayesian
	OR (95% CI)	POR (95% CI)	OR (95%CI)	POR (95%CI)
**Age** (1 year)	1.02 (1.00–1.04)	S1 Fig	1.04 (1.01–1.07)	S2 Fig
**Marital status (%)**				
Married or in civil partnership	1.00	1.00	1.00	1.00
Divorced	1.31 (0.66–2.62)	1.41 (0.64–3.10)	1.42 (0.66–3.09)	1.79 (0.77–3.93)
Never married nor in civil partnership	0.68 (0.44–1.06)	0.60 (0.38–0.96)	0.45 (0.23–0.87)	0.39 (0.16–0.84)
Widowed	1.05 (0.11–10.42)	1.10 (0.11–13.82)	1.67 (0.16–17.37)	3.74 (0.11–341.65)
**Country of birth (%)**				
Luxembourg	1.00	1.00	NA **^e^**	1.00
Portugal	1.26 (0.68–2.33)	1.37 (0.81–2.62)		0.85 (0.36–2.12)
Other EU countries	1.60 (0.94–2.73)	1.56 (0.96–2.47)		0.96 (0.44–1.97)
Non EU countries	2.19 (1.15–4.15)	3.24 (1.61–8.69)		1.64 (0.47–4.94)
**Education level (%)**				
Primary	1.00	1.00	1.00	1.00
Secondary (finish)	1.60 (0.94–2.73)	1.66 (1.06–2.72)	1.82 (0.92–3.62)	2.09 (1.05–3.65)
Tertiary	1.08 (0.61–1.92)	1.04 (0.58–1.72)	0.85 (0.41–1.80)	0.86 (0.34–1.90)
**WRPA** **^a^** **(%)**				
Mostly WRPA	1.00	1.00	1.00	1.00
No mostly WRPA	0.80 (0.50–1.26)	0.76 (0.50–1.30)	1.25 (0.65–2.41)	1.22 (0.54–2.43)
Not working	0.75 (0.39–1.45)	1.21 (0.35–5.31)	1.16 (0.30–4.43)	1.44 (0.30–9.32)
**TRPA** **^b^** **(100 MEP units)**	NA	1.00 (0.98–1.02)	0.98 (0.96–1.01)	0.98 (0.96–1.02)
**APA** **^c^** **(%)**				
APA < 150 min per week	1.00	1.00	1.00	1.00
APA ≥ 150 min per week	0.83 (0.57–1.21)	0.82 (0.53–1.30)	0.63 (0.36–1.09)	0.56 (0.30–1.19)
**MSPA** **^d^** **(%)**				
MSPA < 2 days per week	NA	1.00	1.00	1.00
MSPA ≥ 2 days per week		1.26 (0.71–2.33)	0.73 (0.37–1.45)	0.59 (0.29–1.27)
**Fruit frequency consumption (N)**				
Less than once a day	NA	1.00	NA	1.00
Once or more a day		0.99 (0.69–1.58)		0.75 (0.39–1.38)
**Vegetable frequency consumption (N)**				
Less than once a day	NA	1.00	NA	1.00
Once or more a day		0.83 (0.53–1.38)		1.67 (0.92–2.85)
**Alcohol consumption (%)**				
No drink	NA	1.00	NA	1.00
6 drinks or less a week		1.27 (0.73–2.08)		0.57 (0.30–1.02)
More than 6 drinks a week		1.05 (0.67–1.55)		0.74 (0.41–1.31)
**Self-perceived health (%)**				
Good or very good	1.00	1.00	1.00	1.00
Fair	1.10 (0.65–1.85)	1.17 (0.55–2.13)	2.35 (1.12–4.92)	3.19 (1.58–6.79)
Bad or very bad	4.99 (1.31–19.10)	4.45 (1.02–36.90)	9.74 (2.04–46.55)	15.01 (2.16–98.09)
**Physical pain intensity (%)**				
From low intensity to no pain	NA	1.00	1.00	1.00
Moderate		0.95 (0.51–1.63)	0.86 (0.37–2.00)	0.66 (0.29–1.48)
Severe or very severe		0.98 (0.50–2.49)	1.50 (0.54–4.16)	1.14 (0.40–3.58)
**Sleep duration (h: hours)**				
> 6 h (employed people)		1.00	1.00	1.00
> 6 h (unemployed people)		1.41 (1.00–2.47)	0.86 (0.24–3.11)	3.66 (2.02–8.03)
≤ 6 h (employed people)		0.47 (0.13–1.55)	2.70 (1.51–4.83)	0.69 (0.08–3.72)
≤ 6 h (unemployed people)		0.77 (0.16–3.20)	0.81 (0.13–5.10)	0.41 (0.04–5.41)
**Depression (%)**				
No depression	NA	1.00	NA	1.00
Depression		0.82 (0.52–1.46)		0.66 (0.30–1.36)

^**a**^ WRPA: Work-related physical activity.

^**b**^ TRPA: Transport-related physical activity.

^**c**^ APA: Aerobic physical activity.

^**d**^ MSPA: Muscle- strengthening physical activity.

^**e**^ NA: Not applicable. It means that this variable was not associated with the outcome in the multivariable analysis. Only variables showing *P* < 0.20 in the univariate analyses were considered for inclusion in the multivariable model.

**Table 4:** Multivariable logistic and Bayesian predictive models of overweight and obesity in women, in relation to normal weight (EHES-LUX_2013-2015_).

Women	Overweight (N = 562)		Obesity (N = 378)	
	Logistic	Bayesian	Logistic	Bayesian
	OR (95% CI)	POR (95% CI)	OR (95%CI)	POR (95%CI)
**Age** (1 year)	1.02 (1.00–1.04)	S1 Fig	1.01 (0.99–1.04)	S2 Fig
**Marital status (%)**				
Married or in civil partnership	NA **^e^**	1.00	NA	1.00
Divorced		1.29 (0.75–2.09)		1.32 (0.70–3.02)
Never married nor in civil partnership		1.27 (0.73–2.09)		2.20 (1.24–3.91)
Widowed		0.30 (0.07–0.86)		0.33 (0.07–1.41)
**Country of birth (%)**				
Luxembourg	1.00	1.00	1.00	1.00
Portugal	1.98 (1.08–3.61)	2.44 (1.25–4.43)	1.35 (0.71–2.57)	1.70 (0.86–3.19)
Other EU countries	0.77 (0.48–1.24)	0.82 (0.53–1.23)	0.77 (0.42–1.42)	0.73 (0.40–1.14)
Non EU countries	0.97 (0.52–1.79)	1.00 (0.53–1.85)	0.55 (0.25–1.21)	0.49 (0.19–0.98)
**Education level (%)**				
Primary	1.00	1.00	1.00	1.00
Secondary (finish)	0.97 (0.59–1.61)	1.01 (0.58–1.99)	0.60 (0.34–1.04)	0.58 (0.32–1.07)
Tertiary	0.76 (0.42–1.38)	0.77 (0.41–1.61)	0.33 (0.16–0.68)	0.34 (0.18–0.64)
**WRPA** **^a^** **(%)**				
Mostly WRPA	1.00	1.00	1.00	1.00
No mostly WRPA	0.87 (0.55–1.37)	0.83 (0.52–1.28)	0.87 (0.48–1.56)	0.83 (0.54–1.53)
Not working	1.04 (0.62–1.74)	2.03 (0.90–4.57)	0.88 (0.37–2.07)	0.99 (0.33–2.86)
**TRPA** **^b^** **(100 MEP units)**	0.99 (0.97–1.01)	0.98 (0.96–1.00)	0.98 (0.96–1.01)	0.98 (0.95–1.01)
**APA** **^c^** **(%)**				
APA < 150 min per week	1.00	1.00	1.00	1.00
APA ≥ 150 min per week	0.82 (0.54–1.24)	0.86 (0.52–1.26)	0.50 (0.29–0.84)	0.44 (0.27–0.77)
**MSPA** **^d^** **(%)**				
MSPA < 2 days per week	1.00	1.00	1.00	1.00
MSPA ≥ 2 days per week	0.96 (0.59–1.56)	0.99 (0.60–1.57)	1.02 (0.54–1.93)	1.07 (0.52–2.31)
**Fruit frequency consumption (N)**				
Less than once a day	NA	1.00	NA	1.00
Once or more a day		0.79 (0.49–1.12)		0.80 (0.46–1.29)
**Vegetable frequency consumption (N)**				
Less than once a day	NA	1.00	1.00	1.00
Once or more a day		1.11 (0.78–1.59)	0.82 (0.52–1.29)	0.92 (0.62–1.39)
**Alcohol consumption (%)**				
No drink	NA	1.00	1.00	1.00
6 drinks or less a week		0.70 (0.48–1.05)	0.58 (0.34–0.99)	0.54 (0.36–0.90)
More than 6 drinks a week		0.66 (0.34–1.23)	0.57 (0.29–1.11)	0.51 (0.26–1.04)
**Self-perceived health (%)**				
Good or very good	1.00	1.00	1.00	1.00
Fair	1.11 (0.69–1.78)	1.12 (0.75–1.86)	2.10 (1.23–3.60)	2.24 (1.33–3.73)
Bad or very bad	1.07 (0.41–2.80)	0.73 (0.22–2.37)	1.13 (0.37–3.42)	0.83 (0.30–3.09)
**Physical pain intensity (%)**				
From low intensity to no pain	NA	1.00	1.00	1.00
Moderate		0.94 (0.60–1.54)	0.97 (0.57–1.64)	0.93 (0.49–1.84)
Severe or very severe		1.40 (0.63–3.19)	1.48 (0.66–3.33)	1.65 (0.73–4.42)
**Sleep duration (h: hours)**				
> 6 h (professionally active people)	NA	1.00	1.00	1.00
> 6 h (professionally inactive people)		0.84 (0.51–1.30)	1.69 (0.68–4.21)	1.16 (0.68–1.92)
≤ 6 h (professionally active people)		0.43 (0.16–1.07)	1.31 (0.71–2.41)	1.93 (0.81–4.80)
≤ 6 h (professionally inactive people)		0.43 (0.15–1.37)	0.92 (0.34–2.48)	0.92 (0.33–2.31)
**Depression (%)**				
No depression	NA	1.00	1.00	1.00
Depression		1.16 (0.74–1.89)	1.24 (0.75–2.04)	1.33 (0.76–2.24)

^**a**^ WRPA: Work-related physical activity.

^**b**^ TRPA: Transport-related physical activity.

^**c**^ APA: Aerobic physical activity.

^**d**^ MSPA: Muscle- strengthening physical activity.

^**e**^ NA: Not applicable. It means that this variable was not associated with the outcome in the multivariable analysis. Only variables showing *P* < 0.20 in the univariate analyses were considered for inclusion in the multivariable model.

Women born in Portugal (OR: 1.98 [1.08–3.61]) and men born in a non European country (OR: 2.19 [1.15–4.15]) and perceiving their health as bad or very bad (OR: 4.99 [1.31–19.10]) showed a higher risk to develop overweight. These associations were confirmed through the Bayesian analysis (POR, Women: 2.44 [1.25–4.43]; POR, Men: 3.24 [1.61–8.69] and 4.45 [1.02–36.90], respectively). There were also significant relationships, in men, with age (S1 Fig), the fact to have never been married nor in civil partnership (Protective effect, POR: [0.60 (0.38–0.96)] and with a low educational level (secondary or below) [Deleterious effect: POR: 1.66 (1.06–2.72)], and, in women, with the fact to be widowed (Protective effect, POR: [0.30 (0.07–0.86)] (Table 3, Table 4).

#### Obesity

In men, obesity seems to increase with age (OR: 1.04 [1.01–1.07]), a fair (OR: 2.35 [1.12–4.92]) bad or very bad self-perceived health (OR: 9.74 [2.04–46.55]), sleeping less than 6 h for employed people (OR: 2.70 [1.51–4.83]) and decrease for men who have never been married nor have been in civil partnership (OR: 0.45 [0.23–0.87]).

In men, Bayesian analyses showed that obesity increases with age (S2 Fig), a fair (POR: 3.19 [1.58–6.79], respectively bad or very bad (POR: 15.01 [2.16–98.09]) self-perceived health, sleeping more than 6 hours for unemployed men (POR: 3.66 [2.02–8.03]) and a low educational level (secondary or below) (POR:2.09 [1.05–3.65]); and decreases amongst single men (POR: 0.39 [0.16–0.84]).

In women, both multivariable logistic and Bayesian models showed that obesity decreases with higher education (OR: 0.33 [0.16–0.68], POR: 0.34 [0.18–0.64]), more than 150 minutes of physical activity practice per week (OR: 0.50 [0.29–0.84], POR: 0.44 [0.27–0.77]) and low alcohol consumption (6 drinks or less a week, OR: 0.58 [0.34–0.99], POR: 0.54 [0.36–0.90]), and increases with poor self-perceived health (OR: 2.10 [1.23–3.60], POR: 2.24 [1.33–3.73]). In women, Bayesian analyses showed a protective effect against obesity of a Non European country of birth (POR: 0.49 [0.19–0.98]) and a negative effect of being never been married nor in civil partnership (POR: 2.20 [1.24–3.91]) (Table 4).

### Geographic variation of overweight and obesity between 2013 and 2015 in Luxembourg

**Fig 1:**
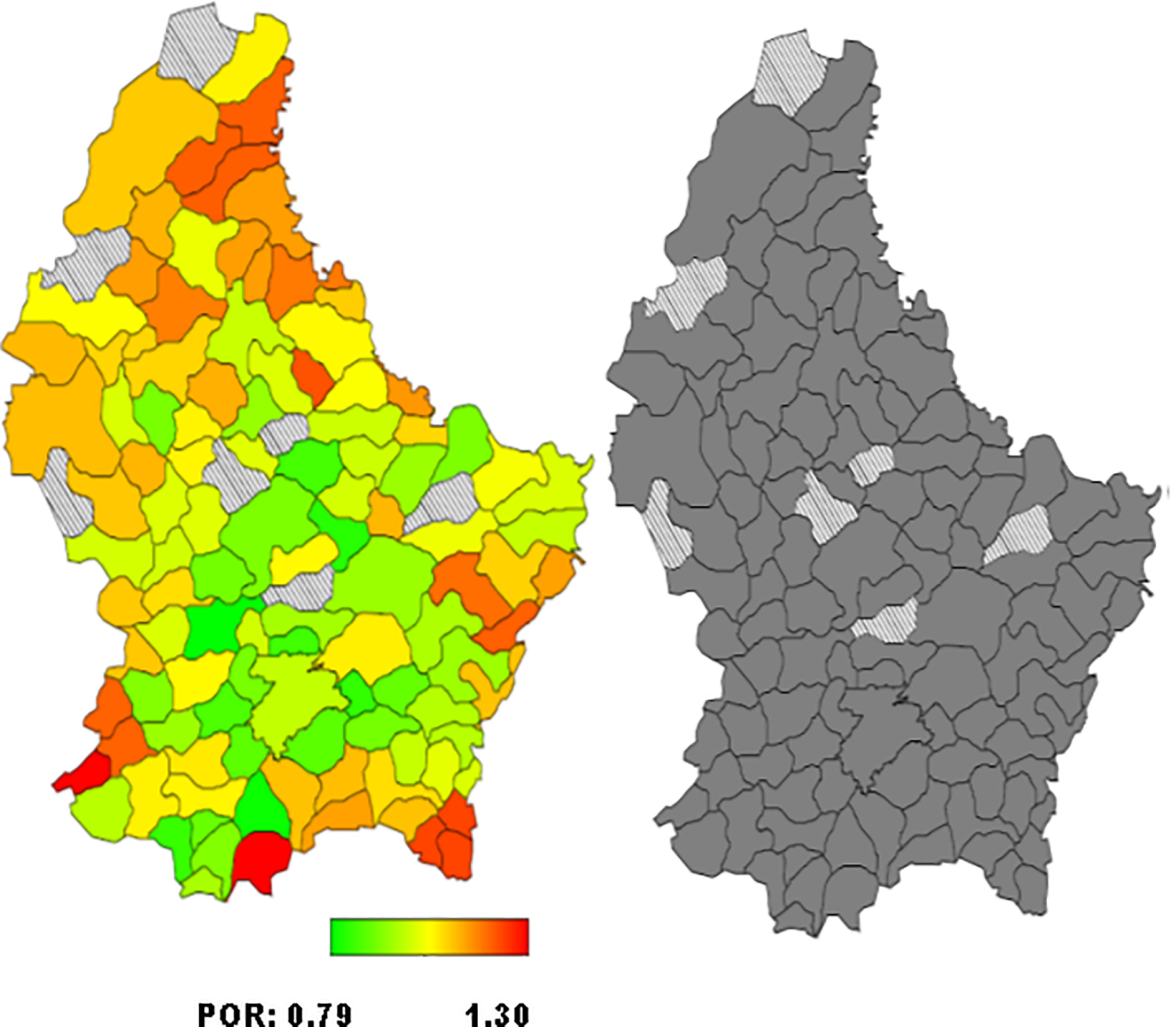
**Left: Adjusted total residual spatial effects for men’s overweight risk, at municipalities-level in Luxembourg in 2013–2015. Shown are the posterior odds ratios. Right: Corresponding posterior probabilities at 80% nominal level (EHES, 2013–2015).** Red coloured–high risk. Green coloured–low risk. Black coloured–significant positive spatial effect. White coloured- significant negative spatial effect. Grey coloured–no significant effect.

**Fig 2:**
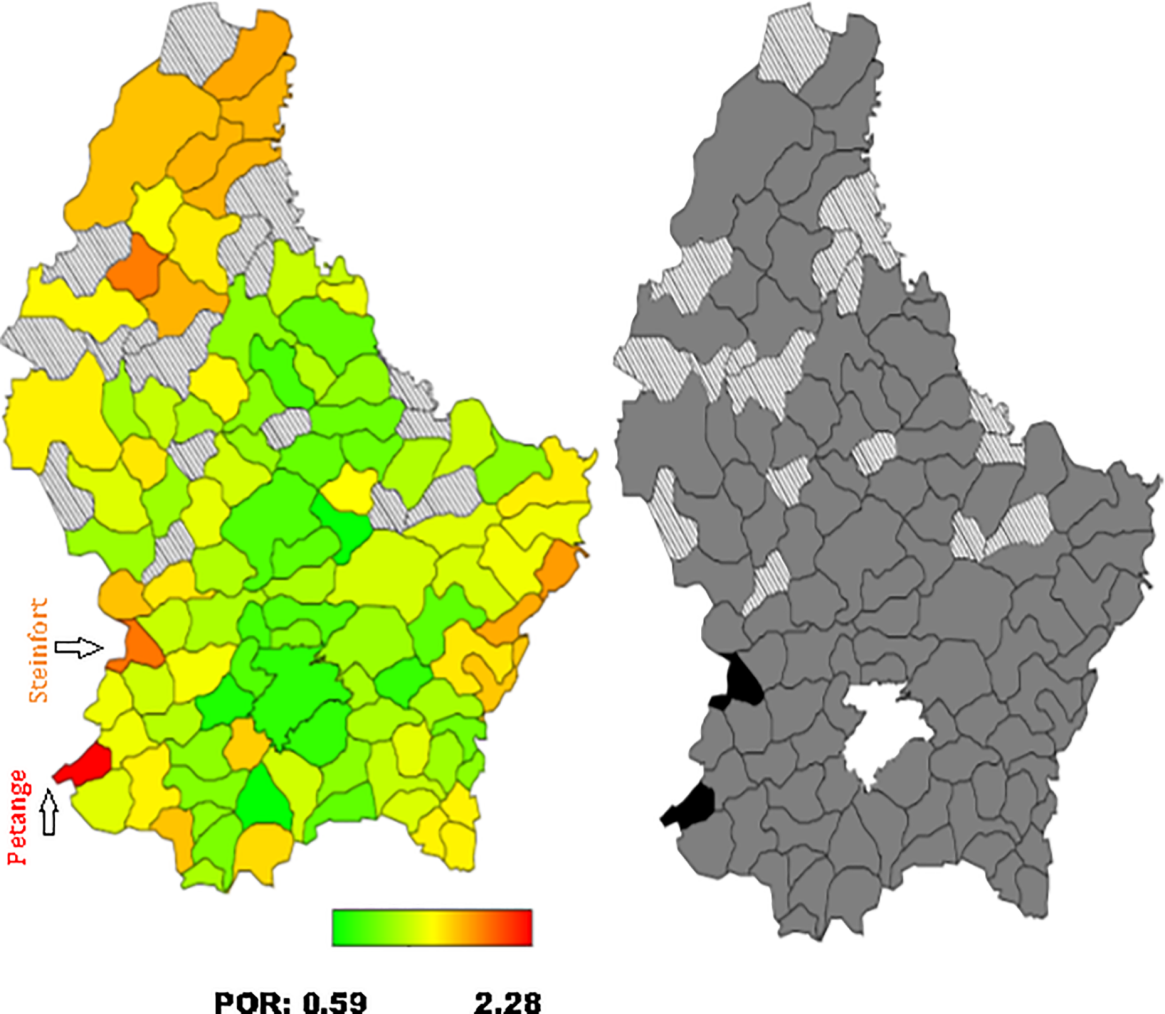
**Left: Adjusted total residual spatial effects for men’s obesity risk, at municipalities-level in Luxembourg in 2013–2015. Shown are the posterior odds ratios. Right: Corresponding posterior probabilities at 80% nominal level (EHES, 2013–2015).** Red coloured–high risk. Green coloured–low risk. Black coloured–significant positive spatial effect. White coloured- significant negative spatial effect. Grey coloured–no significant effect.

**Fig 3:**
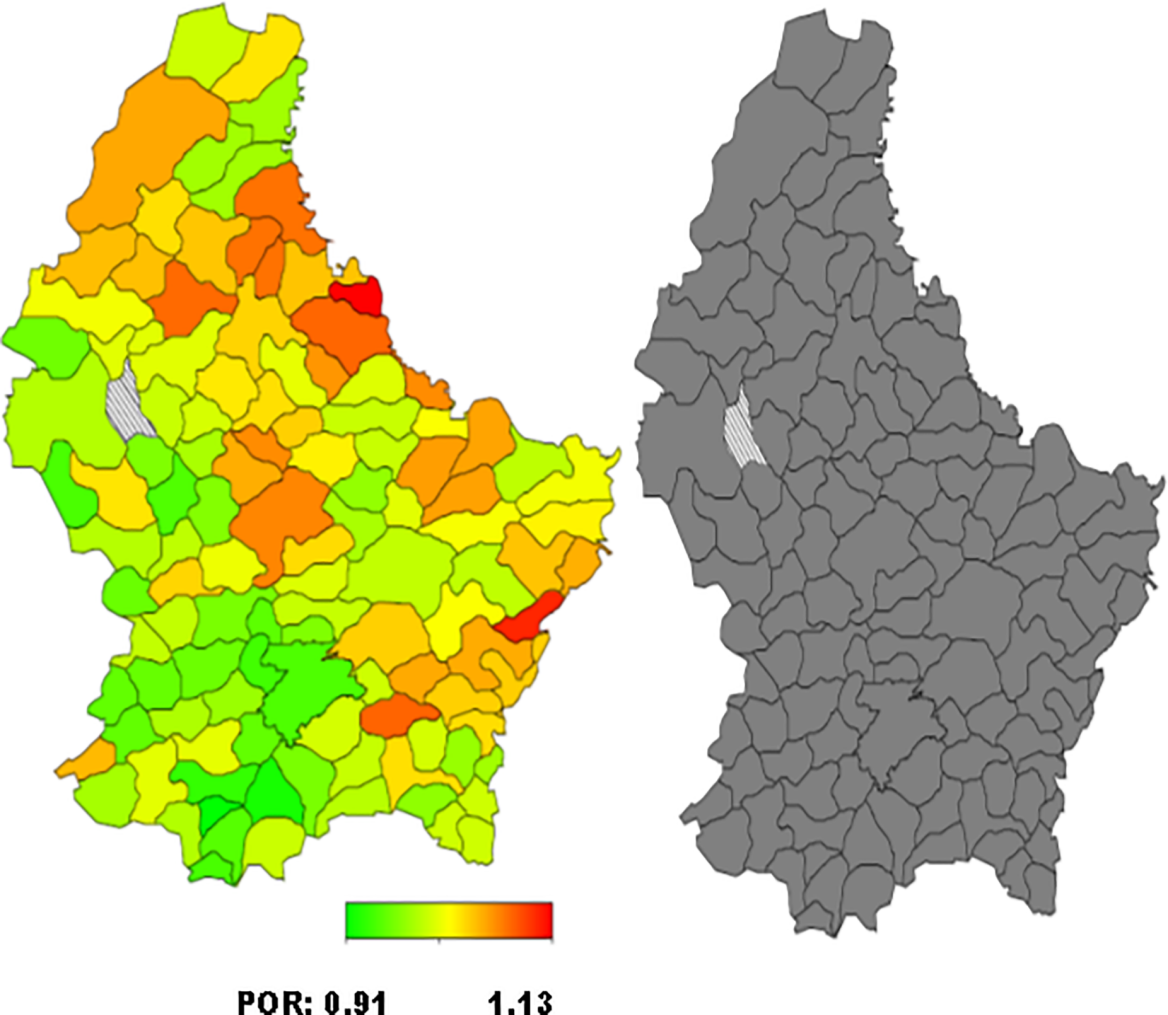
**Left: Adjusted total residual spatial effects for women’s overweight risk, at municipalities-level in Luxembourg in 2013–2015. Shown are the posterior odds ratios. Right: Corresponding posterior probabilities at 80% nominal level (EHES, 2013–2015).** Red coloured–high risk. Green coloured–low risk. Black coloured–significant positive spatial effect. White coloured- significant negative spatial effect. Grey coloured–no significant effect.

**Fig 4:**
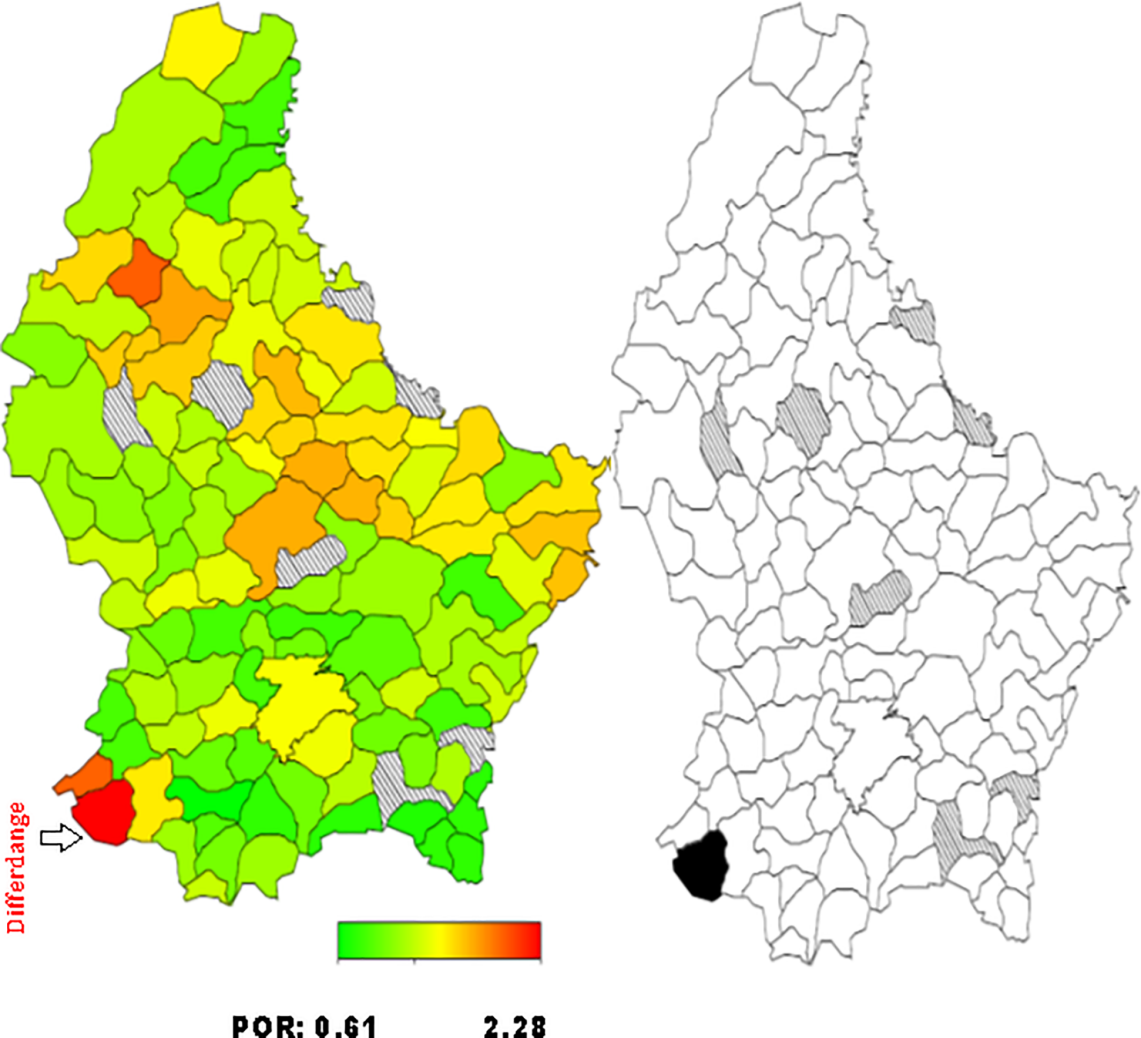
**Left: Adjusted total residual spatial effects for women’s obesity risk, at municipalities-level in Luxembourg in 2013–2015. Shown are the posterior odds ratios. Right: Corresponding posterior probabilities at 80% nominal level (EHES, 2013–2015).** Red coloured–high risk. Green coloured–low risk. Black coloured–significant positive spatial effect. White coloured- significant negative spatial effect. Grey coloured–no significant effect.

Total residual spatial effects for overweight and obesity risk by communes/municipalities adjusted for demographic, socioeconomic, behavioural and health covariates and geographical location, are shown in Figs 1–4.

Higher but not statistically significant posterior odds ratio (POR) of overweight were observed in the South-West for men and in the North- and South-East for women (Figs 1 and 3).

Regarding obesity, the highest and significant POR was located in the South-West for both men (municipalities of Petange and Steinfort) and women (municipality of Differdange) at 80% credible region (CR) (Figs 2 and 4).

## Discussion

The present manuscript represents the first attempt to characterise geographical variation of overweight and obesity in Luxembourg, adjusted for several demographic, socioeconomic, behavioural and health status characteristics, using the most recent data from a nationwide representative sample, with respect to the municipality of residence. In Luxembourg, geographic variation of cardiovascular risk factors has previously been investigated, using data from 2007, and showing in particular a higher overweight/obesity prevalence in some North-East and Centre-East areas [33]. However, the Bayesian approach used by the Authors were only adjusted for gender and age, did neither took into account detailed demographic, socioeconomic, behavioural and health status characteristics, nor distinguished between overweight and obesity which are not especially linked with the same potential health issues [33].

To our knowledge, EHES-LUX_2013-2015_ is the first survey exploring these aspects, also at the European level.

In the present study, the differences observed between logistic (Odd Ratios) and posterior models (Posterior Odd Ratios) showed that as we hypothesised, there is a spatial effect that is not captured by conventional logistic regression models.

In recent years, there has been increasing attention to overweight and obesity mapping adjusted for demographic and socioeconomic characterisics in Africa [21].

Spatial analysis of overweight/obesity performed by Kandala *et al*. [21] at the state-level in Nigeria underlined a highest prevalence of overweight/obesity amongst women in 5 states in the south-eastern areas, as well as in one eastern and one northern states, rather than an exclusive decreasing north-south gradient as showed in some previous studies [17]. These findings were explained by the ongoing urbanisation, westernisation and lifestyle transition process towards more processed high-calorie food and sedentary habits in these states [21].

In our study, after adjustment for demographic, socioeconomic, behavioural and health characteristics, we observed significant geographic variation in the likelihood of developing obesity in both men and women in the South-West of the country, which is in line with the previous work from the same study by Ruiz-Castell *et al*.[26], which focused on hypertension. The authors suggested that the high rates of hypertension in this area may actually be due to the “historical mining and industrial activities, followed by the 1970s’ steel industry crisis which has critically affected the region” [26, 34]. We do agree with Ruiz-Castell *et al*.[26] on the fact that the inhabitants of that geographic area may have been exposed to the double burden of environmental pollutant exposure due to mining and industrialisation and economic vulnerability, both exposures being described as major risk factors for overweight and obesity [35, 36].

Moreover, additional demographic, socioeconomic, behavioural and health-related factors might explain the observed geographic patterns of obesity risk in the present study.

Despite its limited size, Luxembourg shows a very heterogeneous population given the large percentage of immigrants. Two types of immigration exist in the country. There are the highly qualified foreign professionals who live in socioeconomically favourable areas, in particular in the capital, Luxembourg City. There are also less qualified immigrants, living in socioeconomically disadvantaged peripheral regions. The former mining region located in the South-West of Luxembourg, the peripheral region highlighted in the present study, gathers the most economically vulnerable citizens of the country [37].

Socio-cultural factors could also have been involved. Specifically, the process of acculturation related to immigration could play a role in the observed geographic patterns amongst the less qualified immigrants [38–40]. Immigration from low and/or middle towards higher income areas seems to fuel overweight and obesity insofar, as through acculturation, migrant groups may adopt “obesogenic lifestyle habits” in the host country [38]. The process may be the result of several factors such as a rapid epidemiological transition of overnutrition, low physical activity, along with low economic status for migrants and/or easy access to low-quality and high-calorie food [38, 41]. Acculturation process may differ according to gender, which may explain the fact that, in the present survey, men born in Portugal were not impacted [38, 41].

Our findings are in line with the literature regarding the negative impact of a poor self-perceived health on weight gain. Poor health perception has previously been found to be associated with higher rates of cardiometabolic conditions, earlier mortality and, more recently with overweight and obesity, a relationship which seems to be mediated by aging, lack of physical activity, smoking and/or low socioeconomic status [42].

Regarding aging, similar results were observed by Tchicaya *et al*. in 2007 amongst 45–64 years old men in Luxembourg, as well as amongst 45–54 and more than 65 years-old women [11]. Aging is a major risk factor of overweight/obesity, owing to numerous physiological factors such as the menopausal process in women, and the decline of physical activity, muscle mass and metabolic rate in older people [43].

Moreover, the likelihood to develop obesity appeared to be attenuated amongst people living alone. Possible explanations include weight stigmatization in sentimental relationships, also called “the selection hypothesis” and/or “being in the marriage market concept”. The idea behind is that in the Western culture, to get married, single people have to be attractive, therefore slender. Marriage related weight-stigma is mostly experienced by women in the litterature. Our data confirmed this finding in men, but not in women. This obligation disappears after marriage and/or is replaced with “the social obligation hypothesis” advocating a harmonious family life where food is wealthy and caloric [44–46].

Several studies also showed that individuals with lower levels of education are at higher risk of obesity especially in developed countries, which is in line with our findings in women. In middle and high income countries, wealthy women who reached a high level of education show a higher probability to have normal weight [47], while wealth reverses the protective effect of education amongst women living in low income countries [48]. This is possibly linked to a better health education, greater opportunities to access to healthy and non-obesogenic environments, and/or less stressful lifestyle than in socioeconomically deprived areas. Our findings are in line with these studies performed in other countries, and also with the work of Tchicaya *et al*. conducted in 2007 in Luxembourg [11].

Other investigations have pointed out physical activity and low-calorie food consumption as protective factors against weight gain [49]. Unfortunately, people in Westernized countries are regularly exposed to two concomitant phenomena: a plethora of processed foods, available at all times and everywhere, and a sedentary lifestyle resulting of large-scale urbanisation, a quasi-exclusive motorized transportation, less field sports and more screen time. Physical activity is becoming mostly practised as a hobby, under a voluntary approach, with often a busy life leaving little time to be physically active. Eating behaviour has evolved towards a reduction of fruits, vegetables, water and complex carbohydrates consumption, in favour of high fat, sugar-based and high calorie processed food [50, 51]. In the present work, only aerobic physical activity of at least 150 min/week, seems to play a protective role against obesity in women. Inverse relationship between aerobic physical activity (bicycling, dancing, jogging, walking, swimming) and obesity has been previously highlighted in the literature [52, 53]. After adjustment for other risk factors, fruit and vegetable consumption does not seem to impact BMI in our population. Conversely, alcohol consumption restricted to no more than 6 drinks a week appears to attenuate the likelihood to develop obesity in women. In this respect, light to moderate alcohol drinking has previously been shown to reduce the likelihood of obesity [54]. This protective effect seems to be mediated by isohumulones and/or polyphenols found in beer and wine, which may decrease body fat absorption, adipocyte size and weight gain [54].

Short sleep duration was associated with increased obesity in our study, in the subpopulation of employed men. Sleeping less than 6 hours per night has previously been linked to an increased likelihood of obesity [55], and led to recommend a longer sleep duration in recently published guidelines [56].In contrast, we found that unemployed men sleeping more than 6 hours a night appear to be at increased risk of obesity. This is in line with the literature and could be caused by reduced energy expenditure during longer sleeping periods [55].

In contrast with our findings, Alkerwi *et al*. [33] showed an increased overweight/obesity prevalence in the North-East and the Centre-East of the Grand-duché of Luxembourg. These findings were not in line neither with our findings nor with Ruiz-Castell *et al*. regarding hypertension [26]. This may be explained by the fact that Bayesian models used in Alkerwi *et al*. study [33] were only adjusted for gender and age and did not distinguished between overweight and obesity, while our analysis took into account overweight, obesity as well as several demographic, socioeconomic, behavioural and health characteristics. The Authors also performed a secondary analysis of data collected in 2007 and the situation (e.g. demographic, socioeconomic changes, migration) might have changed the last eight years [33].

Finally, this paper provides a comprehensive update of overweight and obesity prevalence amongst adults in Luxembourg, using an objective and direct assessment of BMI. Overweight and obesity impact the vast majority of the population of Luxembougr aged 25–64 (2013–2015) (67.8% in men and 47.7% in women), namely about 182413 people, if extrapolated to the entire population [29]. This places Luxembourg amongst the countries with the highest rates in Europe and worldwide, and implies that the United States of America no longer have such exclusiveness [1]. Our data suggest a much higher prevalence than that published for Luxembourg by the Global Burden of Disease (GBD): i.e. 58% in men and 44.4% in women [1]. This may be caused by methodological differences, and may be related with the fact that BMI data provided by the GBD group were obtained by self-assessment/declaration, which may lead to an under-estimation of body weight [57].

Similar results were found by Tchicaya *et al*. [11] in a survey on “household income and living conditions” in Luxembourg, who also used self-reported BMI. As compared to Tchicaya *et al*. data [11], we found higher prevalences for both overweight (46.77% vs 43.9%) and obesity (21.05% vs 17.9%) in men, and for obesity in women (19.3% vs 17.7%) [11]. The latter discrepancy may also be linked to historical trends over time of overweight and obesity prevalence in Luxembourg (2007 versus 2015). Conversely, obesity rates observed in the present Ehes-Lux_2013-2015_ (20.6%) were close to those observed in the Oriscav-Lux survey (20.9%) performed between 2007 and 2009 in a representative sample of adults living in Luxembourg, which also used a direct assessment of BMI [58].

## Conclusion

The analyses of geographic variations in the prevalence of major chronic conditions, such as overweight and obesity, are an important public health tool to identify “hot spots” and inform allocation of funding for policy and health promotion campaigns. Here we aimed at exploring, for the first time in Luxembourg, potential geographic patterns in overweight/obesity prevalence in the country, adjusted for several demographic, socioeconomic, behavioural and health status characteristics.

Large disparities in the prevalence of overweight and obesity exist between municipalities, with the highest rates of obesity intriguingly found in 3 municipalities situated in the South-West of the country. We hypothesize that a double burden of environmental exposure due to historic mining and industrial activities and past economic vulnaribility may have participated to the higher prevalence of obesity found in these areas, as previously suggested regarding hypertension burden [26]. Other demographic, socioeconomic, behavioural and health covariates could have been involved as well: age, immigration, bad health self-perception, single life, low level of education, low physical activity practice and high alcohol consumption.

EHES-LUX_2013-2015_ showed also high overweight and obesity prevalence, placing Luxembourg amongst the countries with the highest rates. Our findings contribute to the development of context-specific public health policies to tackle the prevalence, correlates and geographic variation of overweight and obesity in Luxembourg.

## Supporting information

S1 Table Univariate logistic predictive models of overweight and obesity in men, in relation to normal weight (EHES-LUX_2013-2015_). WRPA: Work-related physical activity. TRPA: Transport-related physical activity. APA: Aerobic physical activity. MSPA: Muscle- strengthening physical activity.
(DOCX)

S2 Table Univariate logistic predictive models of overweight and obesity in women, in relation to normal weight (EHES-LUX_2013-2015_). WRPA: Work-related physical activity. TRPA: Transport-related physical activity. APA: Aerobic physical activity. MSPA: Muscle- strengthening physical activity.
(DOCX)

S1 Fig Estimated nonparametric trend of women’s and men’s overweight risk by women’s age cohort (left) and men’s age cohort (right) in Luxembourg. Shown is the posterior mean within 80% credible regions [EHES, 2013–2015].
(TIF)

S2 Fig Estimated nonparametric trend of women’s and men’s obesity risk by women’s age cohort (left) and men’s age cohort (right) in Luxembourg. Shown is the posterior mean within 80% credible regions [EHES, 2013–2015].
(TIF)

S1 File Consent form. (PDF)

S2 File EHES questionnaire. (PDF)
