# Supplementary material for: Geographical variation of overweight, obesity and related risk factors: Findings from the European Health Examination Survey in Luxembourg, 2013-2015
Source: PLoS One. 2018 Jun 14;13(6):e0197021. doi: 10.1371/journal.pone.0197021 (PMC6001977; doi:10.1371/journal.pone.0197021)
Supplement: S1 Table — WRPA: Work-related physical activity. TRPA: Transport-related physical activity. APA: Aerobic physical activity. MSPA: Muscle- strengthening physical activity. (DOCX) [file pone.0197021.s001.docx]

| **Men** | **Overweight (N=562)** | **Obesity (N=378)** |
| --- | --- | --- |
|  | **OR (95% CI)** | **OR (95%CI)** |
| **Age** (1 year) | 1.02 (1.00-1.04) | 1.05 (1.03-1.08) |
| **Marital status (%)** | | |
| Married or in civil partnership | 1.00 | 1.00 |
| Divorced | 1.25 (0.64-2.44) | 1.94 (0.95-3.98) |
| Never married nor in civil partnership | 0.58 (0.39-0.87) | 0.41 (0.24-0.71) |
| Widowed | 1.21 (0.11-13.67) | 1.50 (0.09-24.65) |
| **Country of birth (%)** | | |
| Luxembourg | 1.00 | 1.00 |
| Portugal | 1.35 (0.81-2.27) | 1.02 (0.55-1.89) |
| Other EU countries | 1.19 (0.77-1.84) | 0.80 (0.47-1.36) |
| Non EU countries | 1.96 (1.04-3.69) | 0.58 (0.23-1.43) |
| **Education level (%)** | | |
| Primary | 1.00 | 1.00 |
| Secondary (finish) | 1.16 (0.73-1.84) | 1.30 (0.74-2.26) |
| Tertiary | 0.73 (0.47-1.13) | 0.60 (0.35-1.04) |
| **WRPA (%)** | | |
| Mostly WRPA | 1.00 | 1.00 |
| No mostly WRPA | 0.69 (0.46-1.02) | 0.90 (0.55-1.48) |
| Not working | 0.99 (0.56-1.76) | 1.61 (0.82-3.16) |
| **TRPA (100 MEP units)** | 0.99 (0.98-1.01) | 0.99 (0.96-1.01) |
| **APA (%)** | | |
| APA < 150 min per week | 1.00 | 1.00 |
| APA ≥ 150 min per week | 0.73 (0.52-1.04) | 0.40 (0.25-0.64) |
| **MSPA (%)** | | |
| MSPA < 2 days per week | 1.00 | 1.00 |
| MSPA ≥ 2 days per week | 0.94 (0.63-1.41) | 0.43 (0.24-0.77) |
| **Fruit frequency consumption (N)** | | |
| Less than once a day | 1.00 | 1.00 |
| Once or more a day | 0.91 (0.64-1.29) | 0.84 (0.55-1.28) |
| **Vegetable frequency consumption (N)** | | |
| Less than once a day | 1.00 | 1.00 |
| Once or more a day | 0.81 (0.57-1.15) | 1.02 (0.67-1.55) |
| **Alcohol consumption (%)** | | |
| No drink | 1.00 | 1.00 |
| 6 drinks or less a week | 1.17 (0.75-1.84) | 0.74 (0.42-1.30) |
| More than 6 drinks a week | 1.08 (0.70-1.66) | 1.04 (0.63-1.71) |
| **Self-perceived health (%)** | | |
| Good or very good | 1.00 | 1.00 |
| Fair | 1.28 (0.78-2.10) | 2.76 (1.61-4.71) |
| Bad or very bad | 4.96 (1.40-17.56) | 9.00 (2.42-33.49) |
| **Physical pain intensity (%)** | | |
| From low intensity to no pain | 1.00 | 1.00 |
| Moderate | 1.21 (0.73-2.02) | 1.47 (0.81-2.65) |
| Severe or very severe | 1.41 (0.70-2.87) | 2.07 (0.95-4.53) |
| **Sleep duration (h: hours)** | | |
| > 6 h ( employed people) | 1.00 | 1.00 |
| > 6 h ( unemployed people) | 1.06 (0.61-1.83) | 1.77 (0.93-3.34) |
| ≤ 6 h ( employed people) | 1.34 (0.88-2.02) | 2.27 (1.39-3.70) |
| ≤ 6 h ( unemployed people) | 2.15 (0.85-5.38) | 2.75 (0.93-8.13) |
| **Depression (%)** | | |
| No depression | 1.00 | 1.00 |
| Depression | 1.04 (0.65-1.68) | 1.19 (0.68-2.08) |
