# Supplementary material for: Geographical variation of overweight, obesity and related risk factors: Findings from the European Health Examination Survey in Luxembourg, 2013-2015
Source: PLoS One. 2018 Jun 14;13(6):e0197021. doi: 10.1371/journal.pone.0197021 (PMC6001977; doi:10.1371/journal.pone.0197021)
Supplement: S2 Table — WRPA: Work-related physical activity. TRPA: Transport-related physical activity. APA: Aerobic physical activity. MSPA: Muscle- strengthening physical activity. (DOCX) [file pone.0197021.s002.docx]

| **Women** | **Overweight (N=562)** | **Obesity (N=378)** |
| --- | --- | --- |
|  | **OR (95% CI)** | **OR (95%CI)** |
| **Age** (1 year) | 1.03 (1.01-1.04) | 1.04 (1.02-1.06) |
| **Marital status (%)** | | |
| Married or in civil partnership | 1.00 | 1.00 |
| Divorced | 1.49 (0.91-2.43) | 1.70 (0.99-2.92) |
| Never married nor in civil partnership | 0.97 (0.61-1.54) | 0.96 (0.57-1.61) |
| Widowed | 0.49 (0.16-1.50) | 0.76 (0.24-2.36) |
| **Country of birth (%)** | | |
| Luxembourg | 1.00 | 1.00 |
| Portugal | 2.25 (1.34-3.77) | 3.00 (1.74-5.18) |
| Other EU countries | 0.69 (0.45-1.06) | 0.66 (0.40-1.09) |
| Non EU countries | 0.95 (0.55-1.66) | 0.76 (0.38-1.49) |
| **Education level (%)** | | |
| Primary | 1.00 | 1.00 |
| Secondary (finish) | 0.65 (0.42-1.00) | 0.36 (0.22-0.57) |
| Tertiary | 0.39 (0.24-0.62) | 0.14 (0.08-0.25) |
| **WRPA (%)** | | |
| Mostly WRPA | 1.00 | 1.00 |
| No mostly WRPA | 0.66 (0.45-0.98) | 0.49 (0.31-0.76) |
| Not working | 1.09 (0.68-1.74) | 1.18 (0.71-1.95) |
| **TRPA (100 MEP units)** | 0.98 (0.96-1.00) | 0.99 (0.96-1.01) |
| **APA (%)** | | |
| APA < 150 min per week | 1.00 | 1.00 |
| APA ≥ 150 min per week | 0.68 (0.48-0.97) | 0.37 (0.23-0.57) |
| **MSPA (%)** | | |
| MSPA < 2 days per week | 1.00 | 1.00 |
| MSPA ≥ 2 days per week | 0.73 (0.47-1.11) | 0.45 (0.26-0.77) |
| **Fruit frequency consumption (N)** | | |
| Less than once a day | 1.00 | 1.00 |
| Once or more a day | 0.92 (0.65-1.30) | 0.87 (0.59-1.28) |
| **Vegetable frequency consumption (N)** | | |
| Less than once a day | 1.00 | 1.00 |
| Once or more a day | 0.94 (0.66-1.34) | 0.72 (0.48-1.06) |
| **Alcohol consumption (%)** | | |
| No drink | 1.00 | 1.00 |
| 6 drinks or less a week | 0.76 (0.52-1.12) | 0.46 (0.29-0.73) |
| More than 6 drinks a week | 0.77 (0.47-1.27) | 0.65 (0.37-1.14) |
| **Self-perceived health (%)** | | |
| Good or very good | 1.00 | 1.00 |
| Fair | 1.42 (0.92-2.18) | 3.34 (2.15-2.72) |
| Bad or very bad | 1.76 (0.76-4.05) | 3.62 (1.55-8.43) |
| **Physical pain intensity (%)** | | |
| From low intensity to no pain | 1.00 | 1.00 |
| Moderate | 1.00 (0.65-1.54) | 1.73 (1.10-2.72) |
| Severe or very severe | 1.64 (0.90-2.96) | 2.38 (1.25-4.53) |
| **Sleep duration (h: hours)** | | |
| > 6 h ( employed people) | 1.00 | 1.00 |
| > 6 h ( unemployed people) | 1.11 (0.71-1.73) | 2.14 (1.33-3.43) |
| ≤ 6 h ( employed people) | 1.11 (0.69-1.77) | 1.86 (1.10-3.15) |
| ≤ 6 h ( unemployed people) | 1.38 (0.72-2.64) | 2.30 (1.12-4.69) |
| **Depression (%)** | | |
| No depression | 1.00 | 1.00 |
| Depression | 1.11 (0.75-1.65) | 1.95 (1.29-2.95) |
