# Supplementary figures and images for: Geographical variation of overweight, obesity and related risk factors: Findings from the European Health Examination Survey in Luxembourg, 2013-2015
Source: PLoS One. 2018 Jun 14;13(6):e0197021. doi: 10.1371/journal.pone.0197021 (PMC6001977; doi:10.1371/journal.pone.0197021)

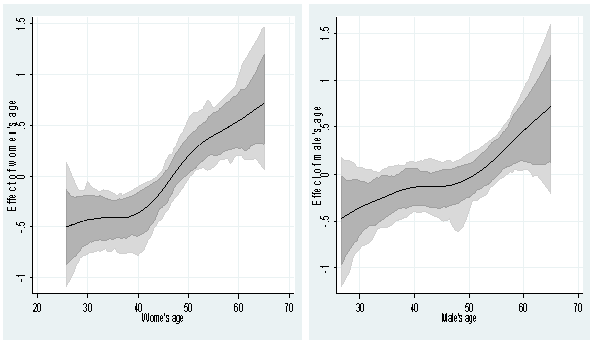

Supplement: S1 Fig — Estimated nonparametric trend of women’s and men’s overweight risk by women’s age cohort (left) and men’s age cohort (right) in Luxembourg. Shown is the posterior mean within 80% credible regions [EHES, 2013–2015]. (TIF) [file pone.0197021.s003.tif]

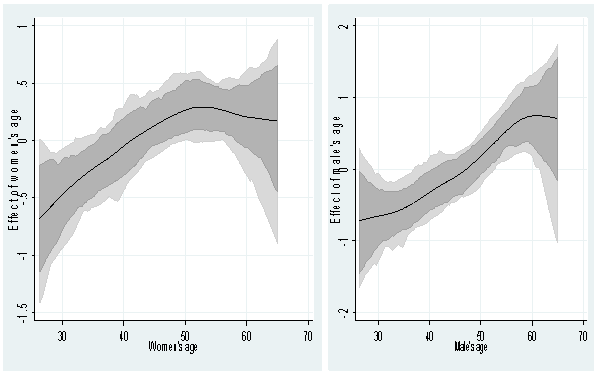

Supplement: S2 Fig — Estimated nonparametric trend of women’s and men’s obesity risk by women’s age cohort (left) and men’s age cohort (right) in Luxembourg. Shown is the posterior mean within 80% credible regions [EHES, 2013–2015]. (TIF) [file pone.0197021.s004.tif]
