# Supplementary material for: Geographical variation of overweight, obesity and related risk factors: Findings from the European Health Examination Survey in Luxembourg, 2013-2015
Source: PLoS One. 2018 Jun 14;13(6):e0197021. doi: 10.1371/journal.pone.0197021 (PMC6001977; doi:10.1371/journal.pone.0197021)
Supplement: S1 File — (PDF) [file pone.0197021.s005.pdf]

LE GOUVERNEMENT
DU GRAND-DUCHÉ DE LUXEMBOURG
Ministère de la Santé

CRP SANTÉ
CENTRE DE RECHERCHE PUBLIC
CENTRE FOR HEALTH STUDIES

INFORMED CONSENT FORM

EUROPEAN HEALTH EXAMINATION SURVEY IN LUXEMBOURG

EHES-LUX

- I have read the invitation letter and information notice mailed to me and understood the sense. I have had the opportunity to ask all the questions I wanted and have had these answered satisfactorily.
- I have been informed about advantages and possible inconveniences directly related to this survey.
- I understand that my participation is voluntary and I can withdraw my consent at any time without any prejudice by informing Dr Andrea Kuemmerle.
- I consent that the health insurance provides to CRP-Santé data concerning my hospitalisations and the drugs that were delivered to me during the year before my participation to the survey.
- I consent that information collected through questionnaires, examinations and laboratory analysis can be used as described in the information notice.
- I am aware of the data storing after the end of the survey for maximum 10 years, in secured place at CRP-Santé.
- I consent that the remaining blood and hair samples can later on be used for public health research as described in the information notice.
- My personal data will be treated in strictly confidential way, as written in the amended law of 2nd August 2002 on personal data protection.
- I agree that the collected data will be analysed in an anonymous manner by the Public Research Centre for Health (CRP-Santé) and the European coordination centre.
- I am aware that publications related to this public health research will concern only scientific results and never identify participants.
- I am aware that the National Committee for Ethic and Research has approved this survey and that the National Commission for Data Protection has been notified.
- I have received a copy of the information notice and of this signed informed consent form.

SIGNATURE PAGE

Place: ___________________________________ Date: ____.____.20____

Name of the participant: ____

Name of the person receiving the informed consent: ________________________________

Signatures: ______________________ ______________________

Participant CRP-Santé Study Nurse

☐ I would like to receive a copy of my results

☐ I would like that Dr (first name, LAST NAME)____

(address) ____

receives a copy of my results.

Persons in charge of EHES-LUX / Contacts

Dr Andrea KUEMMERLE, Project Leader
Centre for Health Studies, CRP-Santé
Tel: (+352) 26 970 818
E-mail: andrea.kuemmerle@crp-sante.lu

Dr Sophie COUFFIGNAL, Medical Investigator
Centre for Health Studies, CRP-Santé
Tel: (+352) 26 970 742
E-mail: sophie.couffignal@crp-sante.lu

Responsibilities

CRP-Santé
Centre for Health Studies
Mrs Marie-Lise LAIR
1a-b Rue Thomas Edison,
L-1445 Luxembourg
Tel: (+352) 26 970 752
E-mail: marie-lise.lair@crp-sante.lu

Ministry of Health
Direction de la Santé
Villa Louvigny
L-2120 Luxembourg
Tel: (+352) 247 855 00
