# Supplementary material for: Geographical variation of overweight, obesity and related risk factors: Findings from the European Health Examination Survey in Luxembourg, 2013-2015
Source: PLoS One. 2018 Jun 14;13(6):e0197021. doi: 10.1371/journal.pone.0197021 (PMC6001977; doi:10.1371/journal.pone.0197021)
Supplement: S2 File — (PDF) [file pone.0197021.s006.pdf]

Dear participant,
Thank you for participating in the EHES-LUX survey.

The results of this study depend primarily on the quality of your answers. As such, it is necessary that your answers to these questions be the best and most honest possible.

If a question is unclear or if you have a doubt about how to respond to it, please ask the study nurse for more information. Please remember that study nurses are bound by professional confidentiality.

At the end of this questionnaire, the study nurse will go through the questions with you to see if you had difficulties in filling in and if necessary help you.

Remember also that all information you provide is protected and strictly confidential. A research number has been assigned to you so that your data may be processed anonymously. Under no circumstances will these data be published or used to identify you.

HOW SHOULD YOU RESPOND TO QUESTIONS?

Before answering, read carefully the question and its response categories.

1) Multiple-Choice questions: Mark with a cross the box ☐ which describes best your answer to the question. For each question tick off one single box, except if you find the instruction "More than one response possible".

Example: *Are you …?*

☐ Male

☒ Female

2) The answer is a number: Make your answer in the boxes shown below.

Example: *How tall are you?*

| 1 | 6 | 8 | cm

3) The answer is a date: Make your answer in the boxes shown below.

Example: *What is your day of birth?*

| 1 | 6 | / | 0 | 2 | / | 1 | 9 | 7 | 5 |

Month Day Year

4) In some cases, we ask you to write the answer (in capital letters).

Example: *What nationality are you?*

| L | U | X | E | M | B | O | U | R | G | I | S | H | | | | |

5) When this symbol comes along "➔", it leads you to the next question.

Example: *Do you smoke?*

☐ Yes, every day

☒ Yes, occasionally

} ➔ **SKIP TO question 4.20**

☐ No, never

If this symbol "➔" doesn't appear after an answer, continue with the next question.

PART 1

HEALTH QUESTIONNAIRE

Start date and time

__ __ / __ __ / __ __ __ __
DD/MM/YYYY

__ __ : __ __
HH:MM

1. PERSONAL INFORMATION

1.01. Are you …?

☐ Male

☐ Female

1.02. What is your date of birth?

__ __ / __ __ / __ __ __ __

Month Day Year

1.03. What nationality are you?

1.04. What is your country of birth?

☐ I do not know

1.05. What is your father's country of birth?

☐ I do not know

1.06. What is your mother's country of birth?

☐ I do not know

1.07. What is your legal marital status?

- [ ] Never married and never in a registered partnership (PACS)
- [ ] Married or in a registered partnership (PACS)
- [ ] Widowed or in a registered partnership that ended with death of partner (neither remarried nor in new registered partnership (PACS))
- [ ] Divorced or in a registered partnership that was legally dissolved (neither remarried nor in new registered partnership (PACS))

1.08. Are you living with someone as a couple (**in a consensual union)**?

I.e. someone to whom you are not legally married or in a registered partnership.

- [ ] Yes
- [ ] No

1.09. How many years did you spend at school or **in full-time study**?

Count from 1st year in Kindergarten.
Include: years referred.
Exclude: evening classes, e-learning, distance learning, etc.

☐☐ years

The next question concerns the ***highest level of education or professional training you have successfully achieved.***

If you are still in secondary school, university or an apprenticeship course*, this question does not pertain to the academic program in which you are currently enrolled, but rather the highest level you have already successfully achieved.*

If you have difficulties in defining your level of education, please refer to the study nurse.

1.10. What is the **highest level** of education or training you **successfully** completed?

- ☐ **Early childhood development, pre-primary education** (maternal pre-primary school)
- ☐ **Primary education** (primary school)
- ☐ **Lower secondary education** (first cycle of the secondary education)
- ☐ **Upper secondary education** (second cycle of the secondary education)
- ☐ **Post-secondary but non-tertiary education** (professional school or preparatory classes to tertiary education if access from secondary level is not direct)
- ☐ Tertiary education; **short-cycle** (advanced professional/technical school or university, 2 successfully completed years)
- ☐ Tertiary education; **bachelor level** or equivalent (advanced professional/technical school or university, 3-4 successfully completed years)
- ☐ Tertiary education; **master level** or equivalent (advanced professional/technical school or university, 5 successfully completed years)
- ☐ Tertiary education; **doctoral level** or equivalent (PhD)

Remarks or description concerning the program or diploma:

Complete title of diploma: ....

....

Year of obtention: ....

Country: ....

1.11. How would you define your current labour status?

- [ ] Carry out a job or profession (including unpaid work for a family business or holding or including an apprenticeship or paid traineeship, etc.)

- [ ] Unemployed
- [ ] Student, further training, unpaid work experience
- [ ] In retirement or early retirement or has given up business
- [ ] Permanently disabled
- [ ] In compulsory military or community service
- [ ] Fulfilling domestic tasks (unpaid)
- [ ] Other inactive status

SKIP TO question 1.14

1.12. In your job, do you work full- or part-time?

- [ ] Full-time
- [ ] Part-time

1.13. Are you self-employed or an employee?

- [ ] Self-employed
- [ ] Employee

→ If you are an employee, are you employed…?

- [ ] With a permanent job/work contract of unlimited duration
- [ ] With a temporary job/work contract of limited duration

1.14. How many persons live in your household?

All persons (**including yourself**)	☐☐	persons
In detail (**per age**):		
Less than or equal to 4 years	☐☐	persons
From 5 years to 13 years	☐☐	persons
From 14 years to 15 years	☐☐	persons
From 16 years to 24 years	☐☐	persons
↳ How many are students?	☐☐	persons
From 25 years to 64 years (**including yourself**)	☐☐	persons
More than or equal to 65 years	☐☐	persons

1.15. In what type of household do you live?

- ☐ One-person household
- ☐ Multi-person household

↳ If multi-person household:

- ☐ Lone parent with child(ren) aged less than 25 years
- ☐ Couple without child(ren) aged less than 25 years
- ☐ Couple with child(ren) aged less than 25 years
- ☐ Couple or lone parent with child(ren) aged less than 25 years and other persons living in household
- ☐ Other type of household

1.16. How many persons aged **16-64 years who are in work** live in your household (**including yourself if concerned**)?

☐☐ persons

1.17. How many persons aged **16-64 years who are unemployed or are economically inactive** live in your household (**including yourself if concerned**)?

☐☐ persons

1.18. What is your household total net monthly income?

*Please include, **for your household**, the income from work, unemployment benefits, guaranteed minimum income, old-age or survivor's benefits, sickness or disability benefits, family/children related allowances, housing allowances, education-related allowance and any other regular benefits, and deduct taxes and welfare contributions.*

*If you do not know the exact amount, **estimate it**.*

☐☐☐☐☐☐ Euros per month

If you do not know the exact amount and you cannot provide an estimate of it → SKIP TO next page

Please indicate your income bracket (net monthly income):

- ☐ Less than 500 Euros
- ☐ From 500 to 999 Euros
- ☐ From 1000 to 1499 Euros
- ☐ From 1500 to 1999 Euros
- ☐ From 2000 to 2499 Euros
- ☐ From 2500 to 2999 Euros
- ☐ From 3000 to 3499 Euros
- ☐ From 3500 to 3999 Euros
- ☐ From 4000 to 4499 Euros
- ☐ From 4500 to 4999 Euros
- ☐ From 5000 to 5999 Euros
- ☐ From 6000 to 6999 Euros
- ☐ From 7000 to 7999 Euros
- ☐ From 8000 to 8999 Euros
- ☐ From 9000 to 9999 Euros
- ☐ From 10000 to 12499 Euros
- ☐ From 12500 to 14499 Euros
- ☐ From 15000 to 19999 Euros
- ☐ From 20000 to 24999 Euros
- ☐ From 25000 to 29999 Euros
- ☐ From 30000 to 34999 Euros
- ☐ From 35000 to 39999 Euros
- ☐ From 40000 to 49999 Euros
- ☐ More than 50000 Euros
- ☐ I do not wish to answer

Working and Living Conditions

The next set of questions is on your working and living conditions. This information will allow us to see if the environmental living and working conditions have an impact on health.

1.19. When has the building you live in been built?

- ☐ Less than 10 years ago
- ☐ Between 10 years and 30 years ago
- ☐ Over 30 years ago
- ☐ I do not know

→ **If the building is over 30 years old**, can you state exactly how old it is?

☐☐☐ years

- ☐ I do not know

1.20. Has any building work been done in your home **over the past three months**?

- ☐ Yes
- ☐ No

1.21. Do you have one or more pets?

- ☐ Yes
- ☐ No

→ **If yes**, do you treat them externally against fleas and ticks?

- ☐ Yes
- ☐ No

1.22. Do you use pesticides (for example herbicides, insecticides, fungicides, etc.) inside your home?

- ☐ Yes
- ☐ No

If yes, for which purpose are you using them? *More than one response possible.*

- ☐ To treat my plants
- ☐ Against flies, mosquitoes, spiders, cockroaches, etc.

1.23. Do you have a garden?

- ☐ Yes
- ☐ No

If yes, do you use pesticides on it?

- ☐ Yes
- ☐ No

1.24. How far **do you live from a heavy traffic road**?

- ☐ Less than 100 m
- ☐ More than 100 m and less than 500 m
- ☐ More than 500 m → **SKIP TO question 1.26**

1.25. What kind of a road is it?

- ☐ Motorway / Highway
- ☐ Main road in town or urban area
- ☐ Main road outside town or urban area
- ☐ Other: ....

1.26. **If you have a job or any professional occupation**, what is your present working address?

....

If you do not have a job or any professional occupation → SKIP TO question 1.31

1.27. **If you have a job or any professional occupation**, how far **do you work from a heavy traffic road**?

- ☐ Less than 100 m
- ☐ More than 100 m and less than 500 m
- ☐ More than 500 m → **SKIP TO question 1.29**

1.28. What kind of a road is it?

- ☐ Motorway/Highway
- ☐ Main road in town or urban area
- ☐ Main road outside town or urban area
- ☐ Other: ....

1.29. How do you travel to work?

If you use more than one means of transportation, please indicate the most frequent or the one you spend most time in.

- ☐ Car
- ☐ Bus
- ☐ Train
- ☐ Bike
- ☐ On foot
- ☐ Other: ....

1.30. How much time do you need **to travel to work and back from work** every day?

☐☐ : ☐☐ per day

Hours Minutes

1.31. Have you worked, **in the last 12 months**, in one or more of the following sectors?

- Public construction and open air
- Industry
- Transportation
- Catering industry and entertainment

☐ Yes

☐ No → **SKIP TO question 2.01 (Health Status)**

If yes, please make it clear below:

More than one response possible.

1. Public construction and open air:

☐ Road maintenance (work with asphalt...)

☐ City maintenance

☐ Maintenance of green spaces

☐ Landscape gardener

☐ Lumberjack, woodcutter

☐ Agriculture, viticulture

☐ Building sites

☐ Traffic and parking checks, inspection

☐ Petrol / gas station (outside in the surrounding of the petrol / gas pump)

☐ Airport (outside in the surrounding of the takeoff runway)

☐ Terraces / cafés close to road

☐ Other: ....

2. Industry:

☐ Mines

☐ Metallurgy: iron, steel, aluminium

☐ Energy (power plant)

☐ Fuel, oil (refinery, tanks)

☐ Recycling

☐ Car: car construction, tire production, car repair shop, vehicle inspection

☐ Chemical / plastics

☐ Incinerator: rubbish, waste, wood

☐ Other: ....

3. Transportation:

☐ Truck driver

☐ Bus driver or taxi driver

☐ Salesman in town, deliverer

☐ Other: ....

4. Catering industry, entertainment:

☐ Cook

☐ Waiter, barman

☐ Swimming pool: maintenance, instructor

☐ Other: ....

2. HEALTH STATUS

The next chapter is about your health.

2.01. How is your health in general?

- ☐ Very good
- ☐ Good
- ☐ Fair
- ☐ Bad
- ☐ Very bad

2.02. Do you have any long-standing illness or long-standing health problem?

The word 'long-standing' refers here to illnesses or health problems which have lasted ***since 6 months****, or are expected to last,* ***at least 6 months****.*

- ☐ Yes
- ☐ No

2.03. **During the last 6 months**, to what extent have you been limited, in activities people usually do, because of a health problem?

- ☐ Severely limited
- ☐ Limited but not severely
- ☐ Not limited at all

Diseases and Chronic Conditions

2.04. Have you **ever** had any of the following diseases or conditions diagnosed by a medical doctor?

High blood pressure (hypertension)	☐ Yes	☐ No
High cholesterol	☐ Yes	☐ No
Diabetes	☐ Yes	☐ No
Coronary heart disease or angina pectoris	☐ Yes	☐ No
Myocardial infarction (heart attack) or chronic consequences of myocardial infarction	☐ Yes	☐ No
Stroke (cerebral haemorrhage, cerebral thrombosis) or chronic consequences of stroke	☐ Yes	☐ No
Stomach or duodenal ulcer	☐ Yes	☐ No
Cirrhosis of the liver or other liver desease	☐ Yes	☐ No
Urinary incontinence, problems in controlling the bladder	☐ Yes	☐ No
Kidney problems	☐ Yes	☐ No
Chronic low back disorder or other chronic back defect	☐ Yes	☐ No
Chronic neck disorder or other chronic neck defect	☐ Yes	☐ No
Rheumatoid arthritis (inflammation of the joints)	☐ Yes	☐ No
Arthrosis (excluding arthritis)	☐ Yes	☐ No
Osteoporosis	☐ Yes	☐ No
Cancer (malignant tumour, also including leukaemia and lymphoma)	☐ Yes	☐ No
Severe headache such as migraine	☐ Yes	☐ No
Chronic anxiety	☐ Yes	☐ No
Depression	☐ Yes	☐ No

If all answers are NO → SKIP TO question 2.06 (Accidents and Injuries)

2.05. **In the past 12 months**, have you had any of the following diseases or conditions?

High blood pressure (hypertension)	☐ Yes	☐ No
High cholesterol	☐ Yes	☐ No
Diabetes	☐ Yes	☐ No
Coronary heart disease or angina pectoris	☐ Yes	☐ No
Myocardial infarction (heart attack) or chronic consequences of myocardial infarction	☐ Yes	☐ No
Stroke (cerebral haemorrhage, cerebral thrombosis) or chronic consequences of stroke	☐ Yes	☐ No
Stomach or duodenal ulcer	☐ Yes	☐ No
Cirrhosis of the liver or other liver desease	☐ Yes	☐ No
Urinary incontinence, problems in controlling the bladder	☐ Yes	☐ No
Kidney problems	☐ Yes	☐ No
Chronic low back disorder or other chronic back defect	☐ Yes	☐ No
Chronic neck disorder or other chronic neck defect	☐ Yes	☐ No
Rheumatoid arthritis (inflammation of the joints)	☐ Yes	☐ No
Arthrosis (excluding arthritis)	☐ Yes	☐ No
Osteoporosis	☐ Yes	☐ No
Cancer (malignant tumour, also including leukaemia and lymphoma)	☐ Yes	☐ No
Severe headache such as migraine	☐ Yes	☐ No
Chronic anxiety	☐ Yes	☐ No
Depression	☐ Yes	☐ No

Accidents and Injuries

2.06. **In the past 12 months,** have you had any of the following types of accidents resulting in injury?

Consider also injuries resulting from poisoning and wilful acts of other persons (aggressions).

Home accident	☐ Yes	☐ No
Leisure accident	☐ Yes	☐ No
Road accident (excluding during the commute to work)	☐ Yes	☐ No
Work accident (excluding during the commute to work)	☐ Yes	☐ No
Road accident during the commute to work	☐ Yes	☐ No

If all answers are NO → SKIP TO question 2.08 (Absence from work due to health problems)

2.07. Did you need medical care as a result of this (these) accident(s)?

Your answers to these questions are linked to your answers to the preceding question. If more than 1 accident occurred, the question refers to ***the most serious in each category****.*

	Yes, I was admitted to hospital or other health facility and stayed overnight	Yes, I was admitted to hospital or other health facility but didn't stay overnight	Yes, I got treated by a doctor or a nurse (not in hospital)	No consultation or intervention was necessary
Accident at home	☐	☐	☐	☐
Leisure accident	☐	☐	☐	☐
Road accident (excluding during the commute to work)	☐	☐	☐	☐
Work accident (excluding during the commute to work)	☐	☐	☐	☐
Road accident during the commute to work	☐	☐	☐	☐

Absence from Work due to Health Problems

2.08. **In the past 12 months**, have you been absent from work due to personal health problems?

Please include all types of diseases, injuries and other health problems that you had and which resulted in your absence from work.

- ☐ Yes
- ☐ No
- ☐ I do not work

→ **SKIP TO question 2.10 (Physical and Sensory Functional Limitations)** (No / I do not work)

2.09. **In the past 12 months**, how many days in total were you absent from work due to personal health problems?

Excluded are: prenatal leave, maternity leave, parental leave, unpaid leave, etc.

☐☐☐ days

Physical and Sensory Functional Limitations

This section is about your general physical health. These questions deal with your ability to do different basic activities. ***Please ignore any temporary problems****.*

2.10. Do you wear glasses or contact lenses?

- ☐ Yes
- ☐ No
- ☐ I am blind or I cannot see at all → **SKIP TO question 2.12**

2.11. Do you have difficulties seeing?

Even when wearing your glasses or contact lenses, if concerned.

- ☐ No difficulty
- ☐ Some difficulty
- ☐ A lot of difficulty
- ☐ I cannot see at all / Unable to do it

2.12. Do you use a hearing aid?

- ☐ Yes
- ☐ No
- ☐ I am profoundly deaf → **SKIP TO question 2.15**

2.13. Do you have difficulties hearing what is said in a conversation with a single person **in a quiet room**?

Even when using your hearing aid, if concerned.

- ☐ No difficulty
- ☐ Some difficulty
- ☐ A lot of difficulty
- ☐ I cannot do it at all / Unable to do it → **SKIP TO question 2.15**

2.14. Do you have difficulties hearing what is said in a conversation with a single person **in a noisier room**?

Even when using your hearing aid, if concerned.

- ☐ No difficulty
- ☐ Some difficulty
- ☐ A lot of difficulty
- ☐ I cannot do it at all / Unable to do it

2.15. Do you have difficulties walking half a kilometer on level ground without the use of any aid?

That would be the length of 5 football fields.

- ☐ No difficulty
- ☐ Some difficulty
- ☐ A lot of difficulty
- ☐ I cannot do it at all / Unable to do it

2.16. Do you have difficulties in walking up or down 12 steps?

That would be one flight of stairs.

- ☐ No difficulty
- ☐ Some difficulty
- ☐ A lot of difficulty
- ☐ I cannot do it at all / Unable to do it

Personal Care Activities

Now we would like you to consider everyday personal care.
Please ignore temporary problems.

2.17. Do you usually have difficulties doing any of these activities without help?

	No difficulty	Some difficulty	A lot of difficulty	Cannot do at all / unable to do
Feeding yourself	☐	☐	☐	☐
Getting in and out of bed or a chair	☐	☐	☐	☐
Dressing and undressing	☐	☐	☐	☐
Using toilets	☐	☐	☐	☐
Bathing or showering	☐	☐	☐	☐

If all answers are NO DIFFICULTY → SKIP TO question 2.21 (Household Activities)

Consider all personal care activities where you have difficulties in doing them without help.

2.18. Do you usually receive help for any of these activities?

☐ Yes, at least for one activity

☐ No → **SKIP TO question 2.20**

2.19. **If yes**, would you need more help?

☐ Yes, at least for one activity

☐ No

→ **SKIP TO question 2.21 (Household Activities)**

2.20. Would you need help?

☐ Yes, at least for one activity

☐ No

Household Activities

Now we would like you to consider some household activities.
Please ignore any temporary problems.

2.21. Do you usually have difficulties doing any of these activities without help?

	No difficulty	Some difficulty	A lot of difficulty	Cannot do at all / unable to do	Never tried it or do not need to do it
Preparing meals	☐	☐	☐	☐	☐
Using the telephone	☐	☐	☐	☐	☐
Shopping	☐	☐	☐	☐	☐
Managing medication	☐	☐	☐	☐	☐
Light housework	☐	☐	☐	☐	☐
Occasional heavy housework	☐	☐	☐	☐	☐
Taking care of finances and everyday administrative tasks	☐	☐	☐	☐	☐

If all answers are NO DIFFICULTY or NEVER TRIED IT OR DO NOT NEED TO DO IT → SKIP TO question 2.25 (Pain)

Consider all household activities where you have difficulties in doing them without help.

2.22. Do you usually have help with one of these activities?

☐ Yes, at least for one activity

☐ No → **SKIP TO question 2.24**

2.23. **If yes**, would you need more help?

☐ Yes, at least for one activity

☐ No

→ **SKIP TO question 2.25 (Pain)**

2.24. Would you need help?

☐ Yes, at least for one activity

☐ No

Pain

*Next questions are about any physical pain you have had **during the past 4 weeks**.*

2.25. Over the **past 4 weeks**, how much physical pain have you had?

- ☐ None
- ☐ Very mild
- ☐ Mild
- ☐ Moderate
- ☐ Severe
- ☐ Very severe

2.26. Over the **past 4 weeks**, how much did pain interfere with your normal work (including work outside the home and housework)?

- ☐ Not at all
- ☐ A little bit
- ☐ Moderately
- ☐ Quite a bit
- ☐ Extremely

Mental Well-Being

*Next questions are about how you feel and how things have been with you **during the past 2 weeks**. For each question, please give the answer that comes closest to the way you have been feeling.*

2.27. Over **the last 2 weeks,** how often have you been bothered by any of the following problems?

	Not at all	Several days	More than half the days	Nearly every day
Little interest or pleasure in doing things	☐	☐	☐	☐
Feeling down, depressed or hopeless	☐	☐	☐	☐
Trouble falling or staying asleep, or sleeping too much	☐	☐	☐	☐
Feeling tired or having little energy	☐	☐	☐	☐
Poor appetite or overeating	☐	☐	☐	☐
Feeling bad about yourself or that you are a failure or have let yourself or your family down	☐	☐	☐	☐
Trouble concentrating on things, such as reading the newspaper or watching television	☐	☐	☐	☐
Moving or speaking so slowly that other people could have noticed. Or the opposite - being so fidgety or restless that you have been moving around a lot more than usual	☐	☐	☐	☐
Thoughts that you would be better off dead, or of hurting yourself in some way	☐	☐	☐	☐

Sleep

2.28. How many hours do you sleep normally at night **when you have to work the next day?**

☐☐ hours

☐ Not applicable (i.e.: early retirement, retirement, unemployed, etc.)

2.29. How many hours do you sleep normally at night **when you do NOT work the next day?**

☐☐ hours

2.30. Have you ever told a doctor or other health professional that you have trouble sleeping?

☐ Yes

☐ No

☐ I do not remember

2.31. Have you ever been told by a doctor or other health professional that you have a sleep disorder?

☐ Yes

☐ No

☐ I do not remember

2.32. Do you have difficulties in sleeping the night through?

☐ Yes

☐ No

2.33. Do you regularly take a nap?

☐ Yes

☐ No → **SKIP TO question 2.35**

2.34. How many minutes per day **on average** do you spend napping?

☐☐☐ minutes

2.35. Do you snore?

- ☐ Yes
- ☐ No
- ☐ I do not know

(No / I do not know) → **SKIP TO question 2.39**

2.36. Your snoring is?

- ☐ Slightly louder than breathing
- ☐ As loud as talking
- ☐ Louder than talking
- ☐ Very loud – can be heard in adjacent room

2.37. How often do you snore?

- ☐ Nearly every day
- ☐ 3-4 times a week
- ☐ 1-2 times a week
- ☐ 1-2 times a month
- ☐ Never or nearly never

2.38. Has your snoring ever bothered other people?

- ☐ Yes
- ☐ No
- ☐ I do not know

2.39. Has anyone noticed that you stop breathing during your sleep?

- ☐ Nearly every day
- ☐ 3-4 times a week
- ☐ 1-2 times a week
- ☐ 1-2 times a month
- ☐ Never or nearly never

2.40. How often do you feel tired or limp after your sleep?

- ☐ Nearly every day
- ☐ 3-4 times a week
- ☐ 1-2 times a week
- ☐ 1-2 times a month
- ☐ Never or nearly never

2.41. During your waking time, do you feel tired, limp or not up to par?

- ☐ Nearly every day
- ☐ 3-4 times a week
- ☐ 1-2 times a week
- ☐ 1-2 times a month
- ☐ Never or nearly never

2.42. Have you ever nodded off or fallen asleep while driving a vehicle?

- ☐ Yes
- ☐ No → **SKIP TO question 2.44**

2.43. **If yes**, how often does this occur?

- ☐ Nearly every day
- ☐ 3-4 times a week
- ☐ 1-2 times a week
- ☐ 1-2 times a month
- ☐ Never or nearly never

2.44. How likely are you to doze off or fall asleep in the following situations in comparison to feeling just tired?

This refers to your usual way of life in recent times. Even if you have not done some of these things recently, try to figure out how they would have affected you. Use the following scale to choose the most appropriate grade for each situation.

	I **never** doze	**Slight chance** of dozing	**Moderate chance** of dozing	**High chance** of dozing
Sitting and reading	☐	☐	☐	☐
Watching TV	☐	☐	☐	☐
Sitting inactive in a public place (e.g. a theatre, a movie theater or a meeting)	☐	☐	☐	☐
As a passenger in a car for an hour without a break	☐	☐	☐	☐
Lying down to rest in the afternoon when circumstances permit	☐	☐	☐	☐
Sitting and talking to someone	☐	☐	☐	☐
Sitting quietly after a lunch without alcohol	☐	☐	☐	☐
In a car, while stopped for a few minutes in traffic	☐	☐	☐	☐

3. HEALTH CARE

Use of Inpatient and Day Care

The next set of questions is about time spent in hospital. All types of hospitals or clinics are included.

If you are a woman and have had a child, the time spent in hospital for giving birth must not be included.

3.01. **In the past 12 months**, have you been admitted in a hospital as an **inpatient**?

*As an **inpatient** means **overnight or longer**.*

Visits to emergency departments or as outpatient only, must not be included *(for example if you go to hospital for a medical consultation only).*

☐ Yes

☐ No → **SKIP TO question 3.04**

3.02. **In the past 12 months**, how many separate stays in hospital as an **inpatient** have you had?

Please include all the stays that ended in this period.

☐☐☐ stays

3.03. **In the past 12 months**, how many nights in total did you spend in hospital as an **inpatient**?

☐☐☐ nights

3.04. **In the past 12 months**, have you been admitted in hospital as a **day patient**?

*As a **day patient** means admitted to hospital for diagnosis, treatment or other types of health care, but not required to remain overnight.*

☐ Yes

☐ No → **SKIP TO question 3.06 (Use of Ambulatory and Home Care)**

3.05. **In the past 12 months**, how many times have you been admitted in hospital as a **day patient**?

☐☐☐ times

Use of Ambulatory and Home Care

The next question is about visits to dentists, orthodontists or other dental care specialists.

3.06. When was **the last time** that you visited a dentist or orthodontist on your own behalf?

On your own behalf means not while accompanying a child, spouse, etc.

- ☐ Less than 6 months ago
- ☐ 6 months to 12 months ago
- ☐ 12 months ago or longer
- ☐ Never

*The next set of questions is about **consultations** with your **general practitioner or family doctor.** Please include visits to your doctor's office as well as home visits and consultations by telephone.*

3.07. When was **the last time** that you consulted a general practitioner or family doctor on your own behalf?

- ☐ Less than 12 months ago
- ☐ 12 months ago or longer
- ☐ Never

(12 months ago or longer / Never) → **SKIP TO question 3.09**

3.08. **In the past 4 weeks ending yesterday**, how many times did you consult a general practitioner or family doctor on your own behalf?

☐☐ times

Next questions are about consultations with ***medical*** *or* ***surgical specialists.***

Include visits to hospital as ***outpatient or emergency departments only****, but do not include contact to doctors while in hospital as an inpatient or day patient.*

Include visits to doctors at the workplace or school.

3.09. When was **the last time** that you consulted a medical or surgical specialist on your own behalf (except dentist or orthodontist)?

☐ Less than 12 months ago

☐ 12 months ago or longer
☐ Never
→ SKIP TO question 3.11

3.10. **In the past 4 weeks**, how many times did you consult a medical or surgical specialist on your own behalf (except dentist or orthodontist)?

☐☐ times

3.11. **In the past 12 months** have you visited on your own behalf a…?

Physiotherapist or kinesiologist	☐ Yes	☐ No
Psychologist, psychiatrist or psychotherapist	☐ Yes	☐ No

The next question is about ***home care services*** *that cover a wide range of health and social services provided to people with health problems at their homes. These services comprise for example:* ***home care service by a nurse or midwife, home help for the housework or for elderly people, meals on wheels or transport service.***

Only services provided by professional health or social workers should be included.

3.12. **In the past 12 months**, have you used or received any home care services?

☐ Yes

☐ No

Medicine Use

3.13. **In the past 2 weeks**, have you used any medicines that were **prescribed to you by a doctor**?

***If you are a woman**, exclude contraceptive pills or hormones used solely for contraception.*

☐ Yes

☐ No → **SKIP TO question 3.16**

3.14. Were these medications against…?

High blood pressure	☐ Yes	☐ No
Lowering blood cholesterol level	☐ Yes	☐ No
Other cardiovascular disease, such as stroke and heart attack	☐ Yes	☐ No
Diabetes	☐ Yes	☐ No
Stomach troubles	☐ Yes	☐ No
Pain in the joints	☐ Yes	☐ No
Pain in the neck or back	☐ Yes	☐ No
Cancer	☐ Yes	☐ No
Headache or migraine	☐ Yes	☐ No
Depression	☐ Yes	☐ No
Tension or anxiety	☐ Yes	☐ No

3.15. **In the past 2 weeks**, have you used other types of medicines **that were prescribed to you**, such as …?

Sleeping tablets	☐ Yes	☐ No
Antibiotics (such as penicillin for example)	☐ Yes	☐ No

3.16. **In the past 2 weeks**, have you used any medicines or herbal medicines or vitamins **not prescribed by a doctor**?

***If you are a woman**, exclude contraceptive pills or hormones used solely for contraception.*

☐ Yes

☐ No → **SKIP TO question 3.18 (Preventive Services)**

3.17. Were these medications or supplements against…?

Pain in the joints	☐ Yes	☐ No
Pain in the neck or back	☐ Yes	☐ No
Headache or migraine	☐ Yes	☐ No
Cold, flu or sore throat	☐ Yes	☐ No
Stomach troubles	☐ Yes	☐ No
Deficiency of vitamins/minerals or weak immune system	☐ Yes	☐ No

Preventive Services

3.18. Have you **ever** been vaccinated against flu?

☐ Yes

☐ No → **SKIP TO question 3.20**

3.19. When was **the last time** that you were vaccinated against flu?

☐☐ / ☐☐☐☐

Month Year

☐ Too long ago (before last year)

3.20. When was **the last time** that your blood pressure was measured by a health professional?

☐ Within the past 12 months

☐ Between 1 year and less than 3 years

☐ Between 3 years and less than 5 years

☐ More than 5 years ago

☐ Never

3.21. When was **the last time** that your blood cholesterol was measured by a health professional?

☐ Within the past 12 months

☐ Between 1 year and less than 3 years

☐ Between 3 years and less than 5 years

☐ More than 5 years ago

☐ Never

3.22. When was **the last time** that your blood sugar was measured by a health professional?

- ☐ Within the past 12 months
- ☐ Between 1 year and less than 3 years
- ☐ Between 3 years and less than 5 years
- ☐ More than 5 years ago
- ☐ Never

3.23. When was **the last time** that you had a faecal occult blood test?

The aim of the test is to detect minor blood loss in the gastrointestinal tract, anywhere from the mouth to the colon.

- ☐ Within the past 12 months
- ☐ Between 1 year and less than 2 years
- ☐ Between 2 years and less than 3 years
- ☐ More than 3 years ago
- ☐ Never

3.24. When was **the last time** that you had a colonoscopy?

It is visual examination of the colon (with a colonoscope) from the cecum to the rectum.

- ☐ Within the past 12 months
- ☐ Between 1 year and less than 5 years
- ☐ Between 5 years and less than 10 years
- ☐ More than 10 years ago
- ☐ Never

Unmet needs for health care

Sometimes, people have problems in getting medical care when they need it. By means of the next questions we would like to check to what extent you were confronted with such problems ***during past 12 months****.*

3.25. **In the past 12 months**, have you experienced delay in getting health care due to…?

	Yes	No	No need for health care
The time needed to obtain an appointment was too long	☐	☐	☐
Distance or transportation problem	☐	☐	☐

3.26. Was there any time **in the past 12 month**s when you needed the following kinds of care but **could not afford it**?

	Yes	No	No need for health care
Medical care	☐	☐	☐
Dental care	☐	☐	☐
Prescribed medicines	☐	☐	☐
Eyeglasses or hearing aids	☐	☐	☐
Mental health care (by a psychologist or a psychiatrist for example)	☐	☐	☐
Dietician	☐	☐	☐

4. HEALTH DETERMINANTS

Weight and height

4.01. How tall are you without shoes?

☐☐☐ cm

4.02. How much do you weigh without clothes and shoes?

***If you are pregnant**, please give your weight before pregnancy.*

☐☐☐,☐ kg

Physical Activity / Exercise

*The next questions are about the time you spend doing different types of physical activity **in a typical week**. Please answer these questions even if you do not consider yourself to be a physically active person.*

Firstly think about the time you spend doing work. Think of work as the things that you have to do such as paid and unpaid work, work around your home like gardening, taking care of family, studying or training.

4.03. When you are **at work**, which of the following best describes what you do?

- ☐ Mostly sitting or standing
- ☐ Mostly walking or tasks of moderate physical effort
- ☐ Mostly heavy labour or physically demanding work
- ☐ Not performing any working tasks

*The next questions **exclude the work-related physical activities** that you have already mentioned. Now we would like to ask you about **the way you usually get to and from places.** For example: to work, to school, for shopping, or to market…*

4.04. In a **typical week**, on how many days do you **walk** for at least **10 minutes continuously** to get to and from places?

☐ days → **If 0 day, SKIP TO question 4.06**

☐ I never do such physical activities → **SKIP TO question 4.06**

4.05. How much time do you spend **walking** to get to and from places on a **typical day**?

☐ 10 - 29 minutes per day

☐ 30 - 59 minutes per day

☐ Between 1 hour to less than 2 hours per day

☐ Between 2 hours to less than 3 hours per day

☐ 3 hours or more per day

4.06. In a **typical week**, on how many days do you **bicycle** for at least **10 minutes continuously** to get to and from places?

Also include here the use of other non- motor-driven means of active transportation such as scooter, roller or skates etc.

☐ days → **If 0 day, SKIP TO question 4.08**

☐ I never do such physical activities → **SKIP TO question 4.08**

4.07. How much time do you spend **bicycling** to get to and from places on a **typical day**?

- ☐ 10 - 29 minutes per day
- ☐ 30 - 59 minutes per day
- ☐ Between 1 hour to less than 2 hours per day
- ☐ Between 2 hours to less than 3 hours per day
- ☐ 3 hours or more per day

*The next questions **exclude the work and transportation activities** that you have already mentioned. Now we would like to ask you about **sports, fitness and recreational activities (leisure)** that cause at least a small increase in breathing or heart rate such as brisk walking, ball games, jogging, cycling or swimming.*

4.08. In a **typical week**, on how many days do you do **sports, fitness or recreational (leisure) activities** for at least 10 minutes continuously?

☐ days → **If 0 day, SKIP TO question 4.10**

☐ I never do such physical activities → **SKIP TO question 4.10**

4.09. How much time in total do you spend on **sports, fitness or recreational activities** in a **typical week**?

☐☐ : ☐☐ per week

Hours Minutes

*The next question includes all physical activities specifically designed to strengthen your muscles such as doing **resistance training or strength exercises** (for example, exercises using weights, elastic bonds, own body weight, doing knee bends (squats), push-ups (press-ups), sit-ups, etc…).*
Include all such activities even if you have mentioned them before.

4.10. In a **typical week**, on how many days do you do physical activities specifically designed to strengthen your muscles such as **doing resistance training or strength exercises**?

☐ days

☐ I never do such physical activities

Nutritional Habits

4.11. What is your diet type?

- ☐ Normal
- ☐ Vegetarian (excluding meat, fish or seafood)
- ☐ Vegan (excluding meat, fish or seafood and no animal origin foods such as eggs, milk, honey, milk products, etc.)
- ☐ Other: ....

4.12. Are you currently on a specific diet?

- ☐ Yes
- ☐ No

→ **If yes**, what type? (for losing weight, gluten-free, lactose-free, etc.)

....

→ **If yes**, for what reason (more than one response possible)?

- ☐ To lower my blood pressure
- ☐ To reduce my cholesterol level
- ☐ To reduce my blood sugar level
- ☐ To lose weight
- ☐ To keep in shape
- ☐ Coeliac disease
- ☐ Intolerance to gluten/dairy products
- ☐ Other, specify: ....

4.13. Do you use spices and/or herbs?

For example: basil, mixed herbs (herbes de Provence), coriander, cumin, etc.

- ☐ Yes, always
- ☐ Yes, from time to time
- ☐ No, never

4.14. Do you use salt and/or stock cubes, Aromat, Maggi **to prepare your meals?**

- ☐ Yes, salt only
- ☐ Yes, salt and other flavorings
- ☐ No, I add nothing

4.15. Do you put salt in your food **before eating**?

For example: salt, Aromat, Maggi, prepared herbs, soy sauce, etc.

- ☐ Yes, always
- ☐ Yes, from time to time
- ☐ No, never

4.16. Do you put sugar in your tea, coffee or yogurt **before consuming**?

- ☐ Yes, always
- ☐ Yes, from time to time
- ☐ No, never

→ **If yes**, what type of sugar or sweetener do you use **mostly**?

- ☐ White sugar
- ☐ Brown sugar
- ☐ Honey, maple syrup, agave syrup
- ☐ An aspartame-based product such as Canderel
- ☐ Stevia

4.17. How often do you eat fruit (Exclude: fruit juice)?

- ☐ Once or more a day
- ☐ 4 to 6 times a week
- ☐ 1 to 3 times a week
- ☐ Less than 1 time a week
- ☐ Never

(4 to 6 times a week / 1 to 3 times a week / Less than 1 time a week / Never) → **SKIP TO question 4.19**

4.18. How many portions of fruit, of any sort, do you eat **each day** (Exclude: fruit juice)?

☐☐ portions → **SEE table below**

One portion of fresh fruit	
**Medium-sized**	One medium fruit, such as one apple, banana, pear, orange, nectarine, or a sharon fruit,
**Small-sized fruit**	For example: two plums, two satsumas, three apricots, two kiwi fruit, seven strawberries, a handful (about 14) of cherries, six lychees, a handful of blueberries.
**Large fruit**	Half a grapefruit or avocado, one slice of papaya, one slice of melon (two-inch slice), one large slice of pineapple, two slices of mango (two-inch slices).
**Fruit salad**	Three heaped tablespoons of fresh fruit salad.

4.19. How often do you eat vegetables or salad (excluding potatoes and vegetable juice)?

- ☐ Minimum 1 time a day
- ☐ 4 to 6 times a week
- ☐ 1 to 3 times a week
- ☐ Less than 1 time a week
- ☐ Never

(4 to 6 times a week / 1 to 3 times a week / Less than 1 time a week / Never) → **SKIP TO question 4.21**

4.20. How many portions of vegetables or salad (excluding: potatoes and vegetable juice) do you eat **each day**?

☐☐ portions → **SEE table below**

One portion of vegetables	
**Green vegetables**	Two broccoli spears, eight cauliflower florets, four heaped tablespoons of cabbage, spinach, spring greens or green beans.
**Cooked vegetables**	Three heaped tablespoons of cooked (e.g. steamed, boiled, microwaved) vegetables such as courgettes, carrots, Brussels sprouts or swede.
**Salad vegetables**	Three sticks of celery, two-inch piece of cucumber, one medium tomato, seven cherry tomatoes.
**Pulses and beans**	Three heaped tablespoons of kidney, cannelloni or butter beans or chick peas. Remember that beans or pulses only count as one of the five day portions.

4.21. Do you consume organic foods?

- ☐ Yes, always
- ☐ Yes, from time to time
- ☐ No, never → **SKIP TO question 4.22**

If yes, what types of organic foods do you consume?

More than one response possible.

- ☐ Fruit
- ☐ Vegetables
- ☐ Fruit or vegetable juices
- ☐ Meat, fish and eggs
- ☐ Meat alternatives (tofu, tempeh, quorn, seitan and products derived from these)
- ☐ Dairy products (to include milk, yogurt, cheese, etc.)
- ☐ Starches like rice, pasta, potatoes, bread and cereals
- ☐ Food oils
- ☐ Prepared dishes (pre-cooked and ready to eat)
- ☐ Snack foods (chocolate, cookies, pastries, crisps, etc.)

4.22. On the average, how often do you eat barbecued food in the summer?

- ☐ Minimum 1 time a day
- ☐ 4 to 6 times per week
- ☐ 1 to 3 times per week
- ☐ 1 to 3 times per month
- ☐ Less than 1 time a month
- ☐ Never → **SKIP TO question 4.24**

4.23. What type of barbecue do you use most frequently?

- ☐ Charcoal
- ☐ Gas
- ☐ Electric
- ☐ Other: ....

4.24. What meals or snacks do you eat **every day**?

If you are neither working nor studying, answer only for « rest days ».

	Work days	Rest days
Breakfast in the morning	☐ Yes ☐ No	☐ Yes ☐ No
Mid-morning snack	☐ Yes ☐ No	☐ Yes ☐ No
Lunch	☐ Yes ☐ No	☐ Yes ☐ No
Afternoon snack	☐ Yes ☐ No	☐ Yes ☐ No
Dinner	☐ Yes ☐ No	☐ Yes ☐ No
After-dinner snack	☐ Yes ☐ No	☐ Yes ☐ No

4.25. Where do you generally take your **lunch** during the week?

Please indicate the place where you most frequently eat at midday.

- ☐ Restaurant
- ☐ Fast food
- ☐ Sandwich shop
- ☐ Cafeteria
- ☐ At home: Prepared dishes, pre-cooked and ready to eat
- ☐ At home: home cooking
- ☐ At work: prepared dishes, pre-cooked and ready to eat
- ☐ At work: home cooking
- ☐ Other: ....

4.26. In general, how many times per week do you eat **your evening meal outside your home**?

☐ time(s)

4.27. In general, how many times per week do you eat **pre-cooked dishes**?

☐☐ time(s)

4.28. Do you do the food shopping?

- ☐ Yes, always
- ☐ Yes, from time to time
- ☐ No, never → **SKIP TO question 4.30 (Smoking)**

4.29. **When you do the shopping**, do you look at the nutritional information on food packaging?

- ☐ Yes, always
- ☐ Yes, from time to time
- ☐ No, never → **SKIP TO question 4.30 (Smoking)**

If yes, does it influence your food purchases?

- ☐ Yes, always
- ☐ Yes, from time to time
- ☐ No, never → **SKIP TO question 4.30 (Smoking)**

If yes, what message on the packaging tends to make you buy a product?

More than one response possible.

- ☐ "Light" or "For diabetics" headings
- ☐ Calorie content and nutritional values
- ☐ List of ingredients
- ☐ Specific product characteristics (for example rich in Omega 3 or low cholesterol)
- ☐ Other: ....

Smoking

The next questions are on tobacco use. They include all tobacco products (cigarettes, cigars, tobacco pipes, etc.)

4.30. Do you smoke?

- ☐ Yes, daily
- ☐ Yes, occasionally

} → **SKIP TO question 4.31 (Smoker)**

- ☐ Not at all

↳ **If you do not smoke**, have you **ever** smoked **daily** for at least one year?

- ☐ Yes → **SKIP TO question 4.35 (Past-smoker)**
- ☐ No → **SKIP TO question 4.37 (Passive Smoking)**

SMOKER SUBSECTION

4.31. On average, how many cigarettes, cigars, tobacco pipes do you smoke **per day**?

☐☐

☐ I smoke occasionally (i.e. not daily)

4.32. What kind of tobacco products do you consume?

Cigarettes	☐ Yes	☐ No
If you smoke cigarettes:		
Manufactured cigarettes	☐ Yes	☐ No
Hand-rolled cigarettes	☐ Yes	☐ No
Cigars	☐ Yes	☐ No
Tobacco pipe	☐ Yes	☐ No
Other products	☐ Yes	☐ No

4.32bis What kind of tobacco product do you **mostly** consume?

Only one answer possible.

☐ Cigarettes (Manufactured cigarettes or / and hand-rolled cigarettes)

☐ Cigars

☐ Tobacco pipe

☐ Other products, specify: ....

If you do not smoke manufactured or self-rolled cigarettes → SKIP TO question 4.34

4.33. How many **cigarettes**, on average, do you smoke **each day**?

☐☐ cigarettes (manufactures or hand-rolled)

☐ I smoke occasionally (not daily) **→ SKIP TO question 4.37 (Passive Smoking)**

4.34. For how many years have you smoked **daily**?

Please include all separate periods of smoking daily. If you do not remember the exact number of years, please give an estimate.

☐☐ years **→ SKIP TO question 4.37 (Passive Smoking)**

PAST-SMOKER SUBSECTION

4.35. For how many years have you smoked **daily?**

Please include all separate periods of smoking daily. If you do not remember the exact number of years, please give an estimate.

☐☐ years

4.36. When did you stop smoking **daily**?

If you have quit smoking several times, give the time when you last stopped smoking daily.

- ☐ Within the past week
- ☐ Between 1 week and less than 1 month
- ☐ Between 1 month and less than 1 year
- ☐ Before 1 year to 5 years
- ☐ More than 5 years ago

Passive Smoking

The next questions are about passive exposure to tobacco smoke indoor, i.e. ***at home, at work and at public places*** *such as bars, café, train stations, etc.*

4.37. How often are you exposed to tobacco smoke **indoors**?

- ☐ Never or almost never
- ☐ Less than 1 hour per day
- ☐ 1 hour or more a day

4.38. Where are you usually exposed to tobacco smoke indoors?

At home	☐ Yes	☐ No
At work	☐ Yes	☐ No
At public places (cafés, bars, train station, …)	☐ Yes	☐ No

Alcohol Consumption

*The following questions are about your use of alcoholic beverages of any kind: that is, **beer, wine, cider, cocktails, premixes, alcopops, long drinks, spirits, liquors, homemade alcohol, etc.***

4.39. Have you **ever** consumed alcohol, apart from a few sips or trials?

☐ Yes

☐ No, not in my whole live → **SKIP TO question 4.51 (Drugs)**

4.40. How **old** were you when you have consumed an alcoholic drink for the **first time** (more than a few sips or samples)?

☐☐ years

4.41. Have you ever felt you should cut down on your drinking?

☐ Yes

☐ No

4.42. Have people annoyed you by criticising your drinking?

☐ Yes

☐ No

4.43. Have you ever felt bad or guilty about drinking?

☐ Yes

☐ No

4.44. Have you ever had a drink **first thing** in the morning to steady your nerves or get rid of a hangover?

- ☐ Yes
- ☐ No

4.45. **In the past 12 months**, how often have you had an alcoholic drink of any kind?

- ☐ Every day or almost every day
- ☐ 5 - 6 days a week
- ☐ 3 - 4 days a week
- ☐ 1 - 2 days a week
- ☐ 2 - 3 days in a month
- ☐ 1 time a month
- ☐ Less than 1 time a month

→ **SKIP TO question 4.50**

- ☐ Not in the past 12 months, as I no longer drink alcohol
- ☐ Never, or a few sips or trials in my live

→ **SKIP TO question 4.51 (Drugs)**

4.46. Thinking of **Monday to Thursday**, on how many of these 4 days do you usually drink alcohol

- ☐ On all 4 days
- ☐ On 3 of the 4 days
- ☐ On 2 of the 4 days
- ☐ On 1 of the 4 days
- ☐ On none of the 4 days → **SKIP TO question 4.48**

In the following questions, one standard alcoholic drink is defined as the following number of glasses:

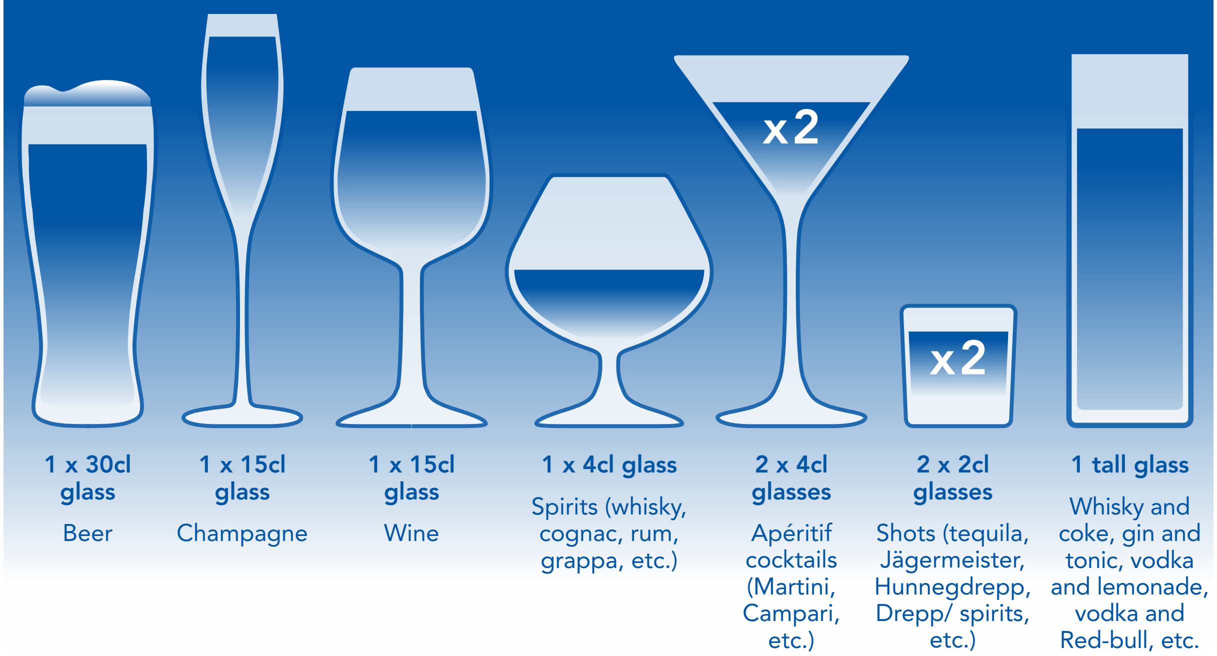

4.47. From **Monday to Thursday**, how many standard drinks (see figure above) do you have on average on such **a day** when you drink alcohol?

- ☐ 16 or more drinks a day
- ☐ 10-15 drinks a day
- ☐ 6 - 9 drinks a day
- ☐ 4 - 5 drinks a day
- ☐ 3 drinks a day
- ☐ 2 drinks a day
- ☐ 1 drink a day
- ☐ 0 drink a day

4.48. Thinking of **Friday to Sunday**, on how many of these 3 days do you usually drink alcohol?

- ☐ On all 3 days
- ☐ On 2 of the 3 days
- ☐ On 1 of the 3 days
- ☐ On none of the 3 days → **SKIP TO question 4.50**

4.49. From **Friday to Sunday**, how many standard drinks do you have on average on **such a day** when you drink alcohol?

- ☐ 16 or more drinks a day
- ☐ 10-15 drinks a day
- ☐ 6 - 9 drinks a day
- ☐ 4 - 5 drinks a day
- ☐ 3 drinks a day
- ☐ 2 drinks a day
- ☐ 1 drink a day
- ☐ 0 drink a day

4.50. **In the past 12 months**, how often have you had **5 or more** standard drinks containing alcohol **on one occasion**?

During a party, a meal, an evening out with friends, alone at home, etc.

- ☐ Every day or almost every day
- ☐ 5 - 6 days a week
- ☐ 3 - 4 days a week
- ☐ 1 - 2 days a week
- ☐ 2 - 3 days in a month
- ☐ 1 time a month
- ☐ Less than 1 time a month
- ☐ Not in the past 12 months
- ☐ Never in my whole life

Drugs

The table below indicates synonyms used for different drugs.

Cannabis	*Grass, green, hay, hash, herb, ganja, blow, blaze, draw, skunk, shit, weed, spliff, dope, buds, pot, skunk, sensimillia, Mary Jane, Reefer*
Ecstasy	*E, MDMA, brownies, pills, smilies*
Amphetamines	*Speed, whizz, uppers, billy, crank, paste*
Cocaine	*C, coke, Charlie, C, snow, nose candy*
Heroin	*Smack, 'H', brown, brown sugar, junk, china white*
Hallucinogenic mushrooms	*Buttons, psilos*
LSD	*Acid, blotter, trip, dot, microdots flash, lucy, L, lightening, purple haze, blaze*
Solvents	*Glue, lighter gas*
New legal substances /products	*Substances/products sold as legal and supposed to imitate illegal drug effects (e.g. powders, pills, tablets, liquids, herbs). Current names given to those products are: legal highs, designer drugs, smart drugs, research chemicals*

4.51. Do you **personally know** people who take the following substances/products?

**Cannabis,** hash, grass	☐ Yes	☐ No
**Ecstasy**	☐ Yes	☐ No
**Amphetamines,** speed	☐ Yes	☐ No
**Cocaine**	☐ Yes	☐ No
**Heroin**	☐ Yes	☐ No
**Hallucinogenic mushrooms**	☐ Yes	☐ No
**LSD,** acid, trip	☐ Yes	☐ No
**Solvents,** glue, lighter gas	☐ Yes	☐ No
**New legal substances/products,** legal highs, designer drugs	☐ Yes	☐ No

4.52. Have you **ever** taken the following substances/products in your life?

**Cannabis,** hash, grass	☐ Yes	☐ No
**Ecstasy**	☐ Yes	☐ No
**Amphetamines,** speed	☐ Yes	☐ No
**Cocaine**	☐ Yes	☐ No
**Heroin**	☐ Yes	☐ No
**Hallucinogenic mushrooms**	☐ Yes	☐ No
**LSD,** acid, trip	☐ Yes	☐ No
**Solvents,** glue, lighter gas	☐ Yes	☐ No
**New legal substances/products,** legal highs, designer drugs	☐ Yes	☐ No

If you answered NO everywhere → SKIP TO question 4.57 (Social Support)

4.53. How **old** were you, when you have taken the following substance(s)/product(s) for **the first time**?

*Answer only the relevant rows, i.e. if answered "**yes**" in the last question.*

**Cannabis,** hash, grass	☐☐
**Ecstasy**	☐☐
**Amphetamines,** speed	☐☐
**Cocaine**	☐☐
**Heroin**	☐☐
**Hallucinogenic mushrooms**	☐☐
**LSD,** acid, trip	☐☐
**Solvents,** glue, lighter gas	☐☐
**New legal substances/products,** legal highs, designer drugs	☐☐

4.54. Have you taken one of these substances/products **over the last 12 months**?

**Cannabis,** hash, grass	☐ Yes	☐ No
**Ecstasy**	☐ Yes	☐ No
**Amphetamines,** speed	☐ Yes	☐ No
**Cocaine**	☐ Yes	☐ No
**Heroin**	☐ Yes	☐ No
**Hallucinogenic mushrooms**	☐ Yes	☐ No
**LSD,** acid, trip	☐ Yes	☐ No
**Solvents,** glue, lighter gas	☐ Yes	☐ No
**New legal substances/products,** legal highs, designer drugs	☐ Yes	☐ No

If you answered NO everywhere → SKIP TO question 4.57 (Social Support)

4.55. Have you taken one of these substances/products **over the last 30 days**?

**Cannabis,** hash, grass	☐ Yes	☐ No
**Ecstasy**	☐ Yes	☐ No
**Amphetamines,** speed	☐ Yes	☐ No
**Cocaine**	☐ Yes	☐ No
**Heroin**	☐ Yes	☐ No
**Hallucinogenic mushrooms**	☐ Yes	☐ No
**LSD,** acid, trip	☐ Yes	☐ No
**Solvents,** glue, lighter gas	☐ Yes	☐ No
**New legal substances/products,** legal highs, designer drugs	☐ Yes	☐ No

If you answered NO everywhere → SKIP TO question 4.57 (Social Support)

4.56. Over **the last 30 days**, on how many days have you taken one of these substances/products?

	20 days or more	10-19 days	4-9 days	1-3 days
**Cannabis,** hash, grass	☐	☐	☐	☐
**Ecstasy**	☐	☐	☐	☐
**Amphetamines,** speed	☐	☐	☐	☐
**Cocaine**	☐	☐	☐	☐
**Heroin**	☐	☐	☐	☐
**Hallucinogenic mushrooms**	☐	☐	☐	☐
**LSD,** acid, trip	☐	☐	☐	☐
**Solvents,** glue, lighter gas	☐	☐	☐	☐
**New legal substances/products,** legal highs, designer drugs	☐	☐	☐	☐

Social Support

The next three questions concern your social contacts.

4.57. How many persons are so close to you that you can count on them if you have serious personal problems?

- ☐ None
- ☐ 1 or 2
- ☐ 3 to 5
- ☐ 6 or more

4.58. How much concern do people show in what you are doing?

- ☐ A lot of
- ☐ Enough
- ☐ Uncertain (this is to say: neither little nor much concern and interest)
- ☐ Little bit
- ☐ Not at all

4.59. How easy is it to get practical help from neighbours if you should need it?

- ☐ Very easy
- ☐ Easy
- ☐ Possible
- ☐ Difficult
- ☐ Very difficult

Provision of Informal Care or Assistance

This section is about the provision of informal care to other people with health problems. ***Exclude any care provided as part of your profession.***

4.60. Do you provide care or assistance to one or more persons suffering from some age problem, chronic condition or infirmity **at least once a week**?

☐ Yes

☐ No → **If you are a WOMAN, SKIP TO question 5.01 (Women's health)**

→ **If you are a MAN, SKIP TO page 71 (Nutrition Questionnaire)**

4.61. Is this person or are these persons…?

If more than 1 category, select the one to whom you are providing the most care.

☐ Member(s) of your family

☐ Not member(s) of your family. Please specify:

- ☐ Neighbour(s)
- ☐ Friends(s)
- ☐ Other(s)

4.62. For how many hours per week do you provide care or assistance?

☐ Less than 10 hours per week

☐ At least 10 hours but less than 20 hours per week

☐ 20 hours per week or more

5. WOMEN'S HEALTH

This chapter is on women health. ***If you are a man, you have finished the health questionnaire. Please go to the nutrition questionnaire page 71.***

5.01. How old were you when you first got your period?

☐☐ years

5.02. Did you have your periods **in the past 3 months**?

☐ Yes → **SKIP TO question 5.04**

☐ No

↳ **If not**, has it been for?

☐ Less than 12 months

☐ More than 12 months

5.03. Is it because..?

More than one response is possible.

☐ You are pregnant

☐ You are breastfeeding

☐ You have a hormonal intrauterine device (Mirena®)

☐ You have a contraceptive implant

☐ You take the contraceptive pill continuously (or a pill that suppresses the periods) or a contraceptive injection

☐ You had a removal of the uterus

☐ You had a removal of the 2 ovaries

☐ You are post-menopausal

☐ Other: ....

....

SKIP TO question 5.05

5.04. If you consider **the last 3 months**, how have your periods been?

☐ Spontaneously regular (cycles from 24 to 32 approximately)

☐ Regular under contraceptive pill or other hormonal treatment

☐ Irregular

5.05. Are you currently using a method to avoid getting pregnant?

☐ Yes

☐ No → **SKIP TO question 5.07**

5.06. **If yes**, could you precise which method(s)?

Contraceptive pill	☐ Yes	☐ No
Intrauterine contraceptive device (IUD)<br>☐ Copper<br>☐ Hormonal (MIRENA®)<br>☐ I do not know	☐ Yes	☐ No
Diaphragm	☐ Yes	☐ No
Spermicide cream or vaginal capsule	☐ Yes	☐ No
Female condom	☐ Yes	☐ No
Male condom	☐ Yes	☐ No
Contraceptive patch (Evra®)	☐ Yes	☐ No
Contraceptive implant (Implanon®)	☐ Yes	☐ No
Contraceptive vaginal ring (Nuvaring®)	☐ Yes	☐ No
Contraceptive injection	☐ Yes	☐ No
Interrupted sexual intercourse (or coitus interruptus)	☐ Yes	☐ No
You do not have any sexual intercourse during the days most at risk (natural methods: Ogino, temperature…)	☐ Yes	☐ No
You or your partner underwent a surgical sterilisation (tubal libation, vasectomy)	☐ Yes	☐ No
Other method:	☐ Yes	☐ No

5.07. Have you **ever** used the contraceptive pill?

☐ Yes

☐ No → **SKIP TO question 5.10**

5.08. How old were you when you began to take the contraceptive pill?

☐☐ years

5.09. **For how many years** have you taken the contraceptive pill?

Please include all separate periods and add them up. Give an estimate if you do not remember exactly.

☐ Less than a year

☐ Between 1 year and 3 years

☐ Between 3 years and 5 years

☐ More than 5 years

5.10. When was **the last time** that you had a cervical smear test?

☐ Within the past 12 months

☐ Between 1 year and less than 2 years

☐ Between 2 years and less than 3 years

☐ More than 3 years

☐ Never

5.11. When was **the last time** that you had mammography (breast X-ray)?

☐ Within the past 12 months

☐ Between 1 year and less than 2 years

☐ Between 2 years and less than 3 years

☐ More than 3 years

☐ Never

5.12. Have you **ever** had an osteodensitometry (measurement of the bone mass)?

☐ Yes

☐ No

5.13. Have you **ever** had any of the following diseases or conditions diagnosed by a medical doctor?

Breast cancer	☐ Yes	☐ No
Uterus cancer	☐ Yes	☐ No

If you answered NO everywhere → SKIP TO question 5.15

5.14. **In the last 12 months**, have you had any of the following diseases or conditions diagnosed by a medical doctor?

Breast cancer	☐ Yes	☐ No
Uterus cancer	☐ Yes	☐ No

5.15. Are you going through the menopause?

☐ Yes

☐ No

☐ I do not know

→ END of the Health Questionnaire, SKIP TO Nutrition Questionnaire page 71

5.16. Are you currently taking any kind of treatment for menopause?

☐ Yes

☐ No

☐ I do not know

→ SKIP TO question 5.19

5.17. What kind of treatment is it?

Hormone replacement therapy	☐ Yes	☐ No
Local vaginal treatment	☐ Yes	☐ No
Non hormonal treatment against flushes	☐ Yes	☐ No
Homoeopathy	☐ Yes	☐ No
Plants	☐ Yes	☐ No
→ **If yes**, is it containing phytoestrogens (e.g. soya)?	☐ Yes	☐ No
Acupuncture	☐ Yes	☐ No
Other treatment, specify:	☐ Yes	☐ No
I do not know	☐	

If you take hormone replacement therapy → CONTINUE
Otherwise → SKIP to question 5.19

5.18. How long have you been taking hormone replacement therapy for menopause?

- ☐ Less than a year
- ☐ Between 1 year and 3 years
- ☐ Between 3 years and 5 years
- ☐ Between 5 years and 10 years
- ☐ More than 10 years

→ END of the Health Questionnaire, SKIP TO Nutrition Questionnaire page 71

5.19. If you are currently not taking any hormone replacement therapy, did you take any in the past?

☐ Yes

☐ No → **END of the Health Questionnaire,**
SKIP TO Nutrition Questionnaire page 71

5.20. **For how many years** did you take hormone replacement therapy?

Please include all separate time periods and add them up. Give an estimate if you do not remember exactly.

☐ Less than 1 year

☐ Between 1 year and 3 years

☐ Between 3 years and 5 years

☐ Between 5 years and 10 years

☐ More than 10 years

5.21. Why did you stop?

☐ Personal choice

☐ Adverse event (I did not tolerate it)

☐ Contra-indication (I suffer from a disease that is a contra-indication)

☐ I do not know

NUTRITION QUESTIONNAIRE

“FOOD FREQUENCY QUESTIONNAIRE”

Over the **past 3 months**, what types of food and drink have you regularly consumed and in what quantities?

The study nurse will explain you how to fill in the questionnaire.

1. Carbohydrates		Never or rarely	1 to 3 times per month	1 to 2 times per week	3 to 5 times per week	Once a day	2 times or more per day	Example of portion size<br>Consult the **photo manual**	Selected photo letter<br>Number of piece(s) or portion(s)
1.	**Sliced white bread,** sandwich bread							reference 1	
2.	**White bread** such as baguette, bread roll, mini-baguette, etc.							reference 2	
3.	**Sliced brown bread (wholemeal, farmhouse, rye)**							reference 1	
4.	**Brown bread** such as baguette, bread roll, etc.							reference 2	
5.	**Savoury biscuits/Rice crackers:** *Krisprolls*, factory-baked toasted bread, *Wasa* crisp bread, wafers, etc.							reference 3	
6.	**Unsweetened breakfast cereals:** *Plain Special K, Fitness original,* muesli flakes, etc.							reference 4	
7.	**Sweetened breakfast cereals:** *Special K/Fitness chocolate, Special K/Fitness fruit, Corn Flakes, Rice Krispies*, honey cereals, chocolate cereals, etc.							reference 4	
8.	**Crunchy muesli** (with chocolate, fruit or dried fruit)							reference 5	

	1. Carbohydrates (CONT'D)	Never or rarely	1 to 3 times per month	1 to 2 times per week	3 to 5 times per week	Once a day	2 times or more per day	Example of portion size<br>Consult the **photo manual**	Selected photo letter<br>Number of piece(s) or portion(s)
**9.**	**Viennese and Danish pastries:** croissants, chocolate croissants, milk bread, raisin rolls (Schneck), 'huit' pastry (Aachtchen), apple turnover, etc.							1 piece	
**10.**	**Shortbread pasty type,** brioche, fruit cake, sponge cake (pound cake, chocolate, Basque, almond, etc.), waffle, shortbread, frangipane, 'Bolo de Arroz', etc.							1 piece	
**11.**	**Cakes such as** éclairs, profiteroles, sweet crêpes, tarts, fruit waffles, "Pasteis de Nata", etc.							1 portion<br>*see reference 6*	
**12.**	Boiled, jacket, steamed **potatoes**							reference 7	
**13.**	**Mashed potatoes (homemade or instant), gnocchi**							reference 8	
**14.**	**Refined cereals:** white pasta, white rice, semolina, vermicelli, etc.							reference 9	
**15.**	**Unrefined cereals:** whole wheat pasta, brown rice, wild rice, Ebly, bulgur wheat, etc.							reference 9	
**16.**	**Fried foods:** all pan-fried or deep-fried potato dishes, gratin dauphinois, potato fritters (Gromperekichelcher)							reference 10	

	**2. Fruit** (excluding fruit juices)	Never or rarely	1 to 3 times per month	1 to 2 times per week	3 to 5 times per week	Once a day	2 times or more per day	Example of portion size	Selected photo letter Number of piece(s) or portion(s)
1.	**Citrus fruit:** orange, clementine, mandarin orange, grapefruit, pomelo, tangerine							1 portion *see reference* 11	
2.	**Red fruit:** strawberries, raspberries, blackberries, blueberries, red currants							reference 12	
3.	**Kiwi**							1 piece	
4.	**Banana**							1 piece	
5.	**Pear, apple, grape, plum, cherry, pineapple, watermelon, lychee**							1 portion *see reference* 13	
6.	**Nectarine, peach, apricot, melon**							1 portion *see reference* 14	
7.	**Tinned fruit in syrup**							reference 15	
8.	**Compote**							1 portion = 1 soup spoon	
9.	**Nuts**							reference 16	
10.	**Unsalted dried fruit:** almonds, peanuts, pistachio nuts, hazelnuts, cashew nuts							reference 17	
11.	**Salted dried fruit:** almonds, peanuts, pistachio nuts, hazelnuts, cashew nuts							reference 17	
12.	**Figs/dates/plums/apricots/raisins (grapes)**							reference 18	

3. Cooked or raw vegetables (excluding vegetable juices)		Never or rarely	1 to 3 times per month	1 to 2 times per week	3 to 5 times per week	Once a day	2 times or more per day	Example of portion size	Selected photo letter<br>Number of piece(s) or portion(s)
1.	**Homemade, frozen or carton soup**							reference 19	
2.	**Packed or tinned soup**							reference 19	
3.	**Peppers**							1 piece	
4.	**Tomatoes:** in any form (including tomato sauce - excluding drinks)							reference 20	
5.	**Carrots**							reference 21	
6.	**Lettuces:** lettuce, crisp salad, mixed salad, endive, rocket, etc.							reference 22	
7.	**Green leafy vegetables:** spinach, celery, chard, etc.							reference 23	
8.	**All types of cabbage:** white cabbage, red cabbage, Brussels sprouts, broccoli, cauliflower, sauerkraut, etc.							reference 24	
9.	**Other vegetables:** aubergine, beetroot, celeriac, chicory, cucumber, courgette, French beans, mangetout, onions, leek, etc.							reference 25	
10.	**Pulses (fresh, frozen or tinned):** flageolet, lentils, chick peas, cannellini beans, peas, sweetcorn							reference 26	
11.	**Avocado**							reference 27	
12.	**Tinned or jarred vegetables** (excluding tomatoes and broad beans): diced mixed vegetables, mushrooms, French beans, peas and carrots, etc.							1 portion = 1 soup spoon	
13.	**Olives:** in any form							reference 28	

4. Meat, poultry, fish, eggs		Never or rarely	1 to 3 times per month	1 to 2 times per week	3 to 5 times per week	Once a day	2 times or more per day	Example of portion size	Selected photo letter<br>Number of piece(s) or portion(s)
1.	**White meat such as:** pork tenderloin, veal (escalope, fillet), chicken breast, turkey poults							reference 29	
2.	**White meat such as:** pork (chop, rib, fillet), veal (chop, minced, breast, hock), chicken leg, whole chicken, rabbit, minced poultry							reference 30	
3.	**White meat such as:** pork (minced, spare rib, pork belly, spirlingue), bacon, pork sausage							reference 31	
4.	**Red meat such as:** beef (sirloin, joint, steak, round of beef), horse, ostrich							reference 29	
5.	**Red meat such as:** beef (rib steak, ribs, minced), lamb (shoulder, leg), carbonnade, duck breast							reference 30	
6.	**Red meat such as:** beef (plate, brisket), lamb (cutlet, breast), mutton, merguez sausage							reference 31	
7.	**Red meat such as:** chipolata, meatloaf, mincemeat, mixed mince, burgers, etc.							reference 31	
8.	**Offal:** liver, kidney, etc.							reference 32	
9.	**Game**							reference 33	

4. Meat, poultry, fish, eggs (CONT'D)		Never or rarely	1 to 3 times per month	1 to 2 times per week	3 to 5 times per week	Once a day	2 times or more per day	Example of portion size	Selected photo letter<br>Number of piece(s) or portion(s)
10.	**Meat preparations:** cordon bleu, Swiss steak, breaded meat, cheese sausage, chicken nuggets, etc.							1 portion = 1 piece = 7 nuggets	
11.	**Unprocessed meat:** cooked ham, chicken and turkey fillet, roast beef, etc.							reference 34	
12.	**Unprocessed smoked meat:** bacon, Ardenne ham, bresaola, etc.							reference 34	
13.	**Processed meat:** black pudding, steak tartare, pâté, salami, Feierstengszalot, dried sausage, saveloy (Lyoner), etc.							reference 35	
14.	**Sandwich fillings:** salads with chicken, fish, salmon, seafood, tuna, etc.							1 portion = 1 soup spoon	
15.	**White fish such as:** cod, sea bream, halibut, whiting, perch, tuna, trout, salted cod, etc.							reference 36	
16.	**Fish such as:** salmon, eel, herring, maatje (young herring)							reference 37	
17.	**Smoked fish:** eel, salmon, trout, or other smoked fish							reference 38	
18.	**Preserved in oil:** tuna, mackerel, sardines, etc.							reference 39	

	4. Meat, poultry, fish, eggs (CONT'D)	Never or rarely	1 to 3 times per month	1 to 2 times per week	3 to 5 times per week	Once a day	2 times or more per day	Example of portion size	Selected photo letter<br>Number of piece(s) or portion(s)
19.	**Fish preparations:** breaded or fried fish, fish fingers, fishcakes, prawn fritters, etc.							reference 40	
20.	**Seafood/Shellfish:** prawn, scampi, crayfish, squid, oyster, etc.							reference 41	
21.	**Mussels:** in any form							reference 42	
22.	**Eggs:** in any form (do not count eggs included in preparations such as cakes, quiches, etc.)							1 egg	
23.	**Vegetable alternatives to cold meat:** Biosmile vegetarian slices, Tartex pâté, Bjorg vegetable terrine, etc.							1 portion<br>*see reference 43*	
24.	**Vegetable alternatives to cold meat, prepared with tofu or Quorn**							1 portion<br>*see reference 44*	
25.	**Vegetable alternatives to meat:** seitan, tofu, fillet, burgers, balls, etc. (Bioline, Vivera, Alpro Soya, Taifun, Quorn, etc.)							1 portion<br>*see reference 45*	
26.	**Vegetable alternative preparations:** burgers, falafel, escalope, cordon bleu, etc. (Bioline, Vivera, Alpro Soya, Taifun, Quorn, etc.)							1 portion<br>*see reference 46*	

	5. Ready-made meals	Never or rarely	1 to 3 times per month	1 to 2 times per week	3 to 5 times per week	Once a day	2 times or more per day	Example of portion size	Selected photo letter<br>Number of piece(s) or portion(s)
1.	**Cod-based dishes:** bacalao, brandade							1 portion	
2.	**Garnished sauerkraut**							reference 47	
3.	**Judd mat Gaardebounen** (Smoked collar of pork with broad beans)							reference 48	
4.	**Pasta** with bolognaise, cheese, béchamel sauce**, stuffed pasta** (cannelloni, ravioli, lasagne, etc.)							reference 49	
5.	**Paella**							reference 50	
6.	**Pizza**							reference 51	
7.	**Quiche with meat or fish, tarte flambée**							reference 52	
8.	**Pâté Riesling (meat pie with Riesling)**							1 portion = 1 tranche	
9.	**Fast food hamburger**							reference 53	
10.	**Deep-fried spring rolls, loempias, nems**							reference 54	
11.	**Cheese croquettes,** cheese pancakes							1 piece	
12.	**Vol-au-vent**							1 portion	
13.	**Pitta**							1 piece	
14.	**Sushi:** Maki, Nigiri, California Roll, Tempura, etc.							1 piece	
15.	***Kniddelen mat Gréiwen/Speck*** (Luxembourg-style wheat dumplings with bacon)							1 portion	
16.	**Stuffed tortilla:** tacos, burritos, wraps							1 piece	
17.	**Stir-fried, Chinese noodles**							reference 55	

	6. Dairy products	Never or rarely	1 to 3 times per month	1 to 2 times per week	3 to 5 times per week	Once a day	2 times or more per day	Example of portion size	Selected photo letter<br>Number of piece(s) or portion(s)
1.	**Plain whole milk**							1 portion = 1 medium glass (150 ml)	
2.	**Plain semi-skimmed milk**							1 portion = 1 medium glass (150 ml)	
3.	**Plain skimmed milk**							1 portion = 1 medium glass (150 ml)	
4.	**Flavoured milk**							1 portion = 1 medium glass (150 ml)	
5.	**Plain and/or light** soya milk or fermented soya dessert							1 portion = 1 medium glass (150 ml)	
6.	Flavoured **soya milk or fermented soya dessert**							1 portion = 1 medium glass (150 ml)	
7.	**Buttermilk, Kefir**							1 portion = 1 medium glass (150 ml)	
8.	**Full-fat yoghurts** (including plain or artificially sweetened **satin or cream yoghurts**), curds							1 portion = 1 small pot	
9.	**Full-fat yoghurts** (including sweetened or fruit **satin or cream yoghurts**)							1 portion = 1 small pot	
10.	Plain or artificially sweetened **low-fat yoghurts (0%)**							1 portion = 1 small pot	
11.	Sweetened or fruit **low-fat yoghurts (0%)**							1 portion = 1 small pot	

6. Dairy products (CONT'D)		Never or rarely	1 to 3 times per month	1 to 2 times per week	3 to 5 times per week	Once a day	2 times or more per day	Example of portion size	Selected photo letter<br>Number of piece(s) or portion(s)
12.	***Yakult, Actimel, Bénécol:*** plain and/or light							1 portion = 1 small bottle	
13.	***Actimel, Benecol-yoghurt:*** flavoured							1 portion = 1 small bottle	
14.	**Fresh cheese:** Quark, cottage cheese, Petit suisse, etc.							reference 56	
15.	**Sweetened fresh cheese:** sweetened maquée, cottage cheese with sugar or fruit, *flavoured Petit Gervais, Danio*, etc.							reference 56	
16.	**Cream cheeses:** *Babybel, Boursin, Vache qui rit, Philadelphia*, etc.							reference 57	
17.	**Low-fat cream cheeses:** *Philadelphia light, Effinesse*, goats' cheese, feta, mozzarella, ricotta, cancoyotte/Kachkéis, etc.							reference 57	
18.	**Soft cheeses:** camembert, brie, Chaumes, etc.							reference 58	
19.	**Low-fat soft cheeses**							reference 58	
20.	**Hard cheeses and blue cheeses:** gouda, chimay, comté, maredsous, passendale, raclette, cheddar, gruyère, parmesan, etc.							reference 59	
21.	**Low-fat hard cheeses and blue cheeses:** gouda light, Leerdamer Ligne, Westlite, gorgonzola light, etc.							reference 59	
22.	**Milk desserts:** bread pudding, custard, rice pudding							1 portion = 1 retail-size pot	

	7. Fats<br>(for spreading, cooking and seasoning)	Never or rarely	1 to 3 times per month	1 to 2 times per week	3 to 5 times per week	Once a day	2 times or more per day	Example of portion size	Selected photo letter<br>Number of piece(s) or portion(s)
1.	**Unsalted butter**							reference 60	
2.	**Lightly salted or salted butter**							reference 60	
3.	**Low-fat or half-fat butter, unsalted**							reference 60	
4.	**Low-fat or half-fat, or lightly salted or salted butter**							reference 60	
5.	**Minarine** such as *Alpro Soya, Effi*, etc.							reference 60	
6.	**Low-fat Minarine** such as *Alpro Soya light, Bécel light, Vitelma light, Primevère léger*, etc.							reference 60	
7.	**Margarine** such as *Fruit d'or tartine et cuisson, Primevère tartiner & cuisson, Planta Classic, Bénécol tartiner et cuire, Bénécol olive*, etc.							reference 60	
8.	**Margarine** such as *Bécel original, Delhaize 35%, Alpro Soya cuire et rôtir liquide light, Bécel cuire et rôtir liquide light*, etc.							reference 60	
9.	**Margarine enriched with omega 3** such as *Bécel oméga 3, Vitelma oméga 3, Planta Good Start, Delhaize oméga 3, Carrefour oméga 3*, etc.							reference 60	

**7. Fats (CONT'D)**<br>(for spreading, cooking and seasoning)		Never or rarely	1 to 3 times per month	1 to 2 times per week	3 to 5 times per week	Once a day	2 times or more per day	Example of portion size	Selected photo letter<br>Number of piece(s) or portion(s)
10.	**Margarine** such as *Bénécol light, Bécel pro-activ, Bertolli pour le pain*, etc.							reference 60	
11.	**Margarine for cooking and roasting** such as *Fama, Solo, Planta, Belolive, Bertolli, Carrefour discount*, etc.							reference 60	
12.	**Margarine for cooking and roasting** such as *Alpro Soya, Bécel, Vitelma, Delhaize, etc.*							reference 60	
13.	**Oil:** olive or peanut							reference 61	
14.	**Oil:** sunflower, corn, grapeseed							reference 61	
15.	**Oil:** rapeseed, soya, walnut							reference 61	
16.	**Mixed oil**							reference 61	
17.	**Fats used outside of meals taken at home**							1 portion	
18.	**'Normal' thick cream, 'normal' double cream**							1 portion =<br>1 soup spoon	

7. Fats (CONT'D)<br>(for spreading, cooking and seasoning)		Never or rarely	1 to 3 times per month	1 to 2 times per week	3 to 5 times per week	Once a day	2 times or more per day	Example of portion size	Selected photo letter<br>Number of piece(s) or portion(s)
19.	Low-fat crème fraîche, low-fat double cream							1 portion = 1 soup spoon	
20.	Soya cream							1 portion = 1 soup spoon	
21.	Low-fat soya cream							1 portion = 1 soup spoon	
22.	Warm sauces: béarnaise, béchamel, pepper sauce, gravy, etc.							1 portion = 1 soup spoon	
23.	Cold sauces: mayonnaise, cocktail, béarnaise, garlic mayonnaise, etc.							1 portion = 1 soup spoon	
24.	Ready-made dressing: low-fat salad dressings							1 portion = 1 soup spoon	
25.	Ketchup							1 portion = 1 soup spoon	
26.	Mustard							1 portion = 1 teaspoon	
27.	Pesto							1 portion = 1 soup spoon	

	8. Miscellaneous	Never or rarely	1 to 3 times per month	1 to 2 times per week	3 to 5 times per week	Once a day	2 times or more per day	Example of portion size	Selected photo letter<br>Number of piece(s) or portion(s)
1.	**Jam, honey, Liege syrup**							reference 62	
2.	**Low-calorie jam**							reference 62	
3.	**Chocolate spread/Chocolate flakes**							reference 63	
4.	**White or milk chocolate:** bars, all sorts of fillings (praline, cream, hazelnuts, etc.)							1 portion = 1 pre-packaged bar = 4 pralines	
5.	**Dark chocolate:** bars, all sorts of fillings (praline, cream, hazelnuts, etc.)							1 portion = 1 pre-packaged bar = 4 pralines	
6.	**Peanut butter**							reference 63	
7.	**Biscuits** (such as *Petit Beurre, Sultana, Grany, Pim's, Choco As*, etc.)							reference 64	
8.	**Chocolate-flavoured snacks** (such as *Cent Wafers, Cha-Cha, Choco Prince, Twix, Snickers, cookie*, etc.), speculoos, wafers, etc.							1 portion = 1 piece = 4 small speculoos	
9.	**Ice creams:** vanilla, chocolate, fruit ice creams							1 portion = 2 scoops	

	8. Miscellaneous (CONT'D)	Never or rarely	1 to 3 times per month	1 to 2 times per week	3 to 5 times per week	Once a day	2 times or more per day	Example of portion size	Selected photo letter<br>Number of piece(s) or portion(s)
10.	**Sorbets or ice lollies**							1 portion =<br>2 scoops =<br>1 ice lolly	
11.	**Filled ice creams: i**ce creams in cornets, on sticks, in a dish (white lady, Brazilian, etc.), Cornetto							1 piece	
12.	**Chantilly, whipped cream**							reference 65	
13.	**Ice cream desserts:** liégeois, mousse, viennoise, etc.							1 portion =<br>1 retail-size pot	
14.	**Aperitif biscuits/Crisps**							reference 66	
15.	**Salted popcorn**							1 portion =<br>1 handful	
16.	**White or brown sugar** (in cubes, loose) for coffee, tea, milk, yoghurt, fromage frais, etc.							1 portion =<br>1 sugar cube =<br>1 teaspoon	
17.	**Boiled sweets (candies) /gums**							1 piece	
18.	**Cocoa powder** (to dilute in milk)							1 portion = 1 teaspoon	

	9. Drinks	Never or rarely	1 to 3 times per month	1 to 2 times per week	3 to 5 times per week	Every day	Indicate the quantities consumed See references 67 and 68	Examples: Water: 1,500 ml; Coffee: three 250 ml cups = 750 ml
1.	**Plain still water, indicate the brand of water:** .... ☐ plastic bottle ☐ glass bottle ☐ can ☐ tap **(*)**						Indicate the quantity **in ml**	
2.	**Plain sparkling water, indicate the brand of water:** .... ☐ plastic bottle ☐ glass bottle ☐ can ☐ tap **(*)**						Indicate the quantity **in ml**	
3.	**Coffee:** normal or decaffeinated						Indicate the quantity **in ml**	
4.	**Tea**						Indicate the quantity **in ml**	
5.	**Substitutes:** herbal tea, chicory, etc.						Indicate the quantity **in ml**	
6.	**Fresh fruit juice**						Indicate the quantity **in ml**	
7.	**Fruit juice** in a can, a bottle, or carton (excluding nectar drinks) ☐ plastic bottle ☐ glass bottle ☐ can ☐ carton **(*)**						Indicate the quantity **in ml**	
8.	**Vegetable juice** in a can, a bottle, or carton ☐ plastic bottle ☐ glass bottle ☐ can ☐ carton **(*)**						Indicate the quantity **in ml**	
9.	**Sweetened drinks:** lemonades, colas, water with fruit cordials (Teisseire cordial), nectars, sweetened flavoured waters, etc. ☐ plastic bottle ☐ glass bottle ☐ can ☐ carton **(*)**						Indicate the quantity **in ml**	
10.	**Light drinks:** light lemonades, light colas, flavoured waters with natural or artificially sweetened flavours, etc. ☐ plastic bottle ☐ glass bottle ☐ can ☐ carton **(*)**						Indicate the quantity **in ml**	

(*) several replies possible

	9. Drinks (CONT'D)	Never or rarely	1 to 3 times per month	1 to 2 times per week	3 to 5 times per week	Every day	Indicate the quantities consumed See references 67 and 68	Examples: Water: 1,500 ml; Coffee: three 250 ml cups = 750 ml
11.	**Low-alcohol beers:** low-alcohol beer<br>☐ plastic bottle ☐ glass bottle ☐ can (*)						Indicate the quantity **in ml**	
12.	**Light beers:** pils, table beers, white beers, etc.<br>☐ plastic bottle ☐ glass bottle ☐ can (*)						Indicate the quantity **in ml**	
13.	**Strong beers:** trappist, abbey beer, etc.<br>☐ plastic bottle ☐ glass bottle ☐ can (*)						Indicate the quantity **in ml**	
14.	**White, rosé wines**						Indicate the quantity **in ml**	
15.	**Red wines**						Indicate the quantity **in ml**	
16.	**Sparkling drinks:** sparkling wines, ciders, Champagnes, etc.						Indicate the quantity **in ml**	
17.	**Aperitif drinks:** aniseed aperitif drink, port, Martini, sherry, Picon, etc.						Indicate the quantity **in ml**	
18.	**Spirits such as:** Amaretto, Bailey's, Get 27, Malibu, Passoã, Pisang, etc.						Indicate the quantity **in ml**	
19.	**Alcohols and spirits:** whisky, brandy, gin, rum, vodka, eau-de-vie, genièvre, etc.						Indicate the quantity **in ml**	
20.	**Energy drinks**<br>☐ plastic bottle ☐ glass bottle ☐ can (*)						Indicate the quantity **in ml**	
21	**Pre-mixed:** Bacardi Breezer, Smirnoff Ice, etc.<br>☐ plastic bottle ☐ glass bottle ☐ can (*)						Indicate the quantity **in ml**	

(*) several replies possible

The first part of the visit you have already placed behind.

Thank you for your contribution.
Now we go on to the 2nd part of the visit.

PART 2

QUESTIONNAIRE MEDICAL EXAMINATION

To fill in by the study nurse

INTERVIEW

1. GENERAL INFORMATION

1.01. Appointment place

- ☐ CRP-Santé
- ☐ CHEM Esch
- ☐ Centre Pontalize
- ☐ Home of participant
- ☐ Other: ……………………..

1.02. Initial letters of the study nurse who performs the measurements

☐☐☐

1.03. Investigation date and time

☐☐ / ☐☐ / ☐☐☐☐
DD MM YYYY

☐☐ : ☐☐
HH:MM

Check of the participant's address :

1.04. In what town do you live in?

☐☐☐☐☐☐☐☐☐☐☐☐☐☐☐☐

If the participant is a woman:

1.05. Are you pregnant?

- ☐ No
- ☐ Yes, weeks of pregnancy: ☐☐
- ☐ Not applicable

1.06. **If the participant is employed or exercises a profession:**

This question refers to paid work. If you have several occupations, consider the ***main one****.*
State the exact designation of your profession*, i.e. do not indicate electrician, but rather electrical contractor ; instead of saleswoman indicate saleswoman for shoes.*

What is your current profession? **Describe your main task accurately!**

....

....

Please do not fill in this section!

ISCO-08

☐☐

(nn)

☐ Do not know, refusal

☐ Not applicable

1.07. Using the most precise terms possible to describe the economic activity of your company?

Here we are considering the workplace and not the entire company.
If you are employed by a temporary agency*, please indicate the business of the company in which you are employed, not the temporary agency.*

....

....

Please do not fill in this section!

NACE Rev.2

☐

(A)

☐ Do not know, refusal

☐ Not applicable

2. MEDICATION

2.01. Do you currently take medications?

☐ Yes

☐ No → **SKIP TO question 3.01 (Conditions of the eyes)**

2.02. Did you bring your medicine packages or a regulation with you?

☐ Yes

☐ No

2.03. Would you allow me to have a look and write down which medicines you are taking, the dosage and the duration of the treatment ?

Do not forget to check: *tablets, capsules, solutions, inhalations, eye drops, creams, patches, injections, infusion, vitamins, homoeopathic or phytotherapy, etc.*

☐ Refusal ☐ Forgotten

In case the person forgot the drug details or in case of missing data, propose to call back.

Example: Aspirine tablet 500mg, dosage 1g 3x/day during 5 days

Brand name of drug	Galenic form	Dosage	Number of dosage units	Frequency	Duration
i.e. ASPIRINE	TABLET	500 mg	2	1-1-1-0	Since 5 days

Brand name of drug	Galenic form	Dosage	Number of dosage units	Frequency	Duration
i.e. ASPIRINE	TABLET	500 mg	2	1-1-1-0	Since 5 days

3. CONDITIONS OF THE EYES

3.01. Have you **ever** had any of the following diseases or conditions diagnosed by a medical doctor?

Glaucoma	☐ Yes	☐ No
Retinopathy	☐ Yes	☐ No
Macular degeneration	☐ Yes	☐ No
Cataract	☐ Yes	☐ No

If all suggestions are NO → GO TO question 4.01 (Thyroid Health)

3.02. **In the past 12 months**, have you had one of the following diseases or conditions?

Glaucoma	☐ Yes	☐ No
Retinopathy	☐ Yes	☐ No
Macular degeneration	☐ Yes	☐ No
Cataract	☐ Yes	☐ No

If all suggestions are NO → GO TO question 4.01 (Thyroid Health)

3.03. **In the past 2 weeks**, have you taken medicines, prescribed by a doctor, for the following diseases?

Glaucoma	☐ Yes	☐ No
Macular degeneration	☐ Yes	☐ No

4. THYROID HEALTH

4.01. Have you **ever** had any thyroid problems diagnosed by a medical doctor?

☐ Yes

☐ No → **GO TO question 4.08**

4.02. **In the past 12 months**, have you had any thyroid problems?

☐ Yes

☐ No → **GO TO question 4.08**

4.03. Have you **ever** been operated for a thyroid problem?

☐ Yes

☐ No → **GO TO question 4.04**

If yes, have you had any surgery **in the last 12 months**?

☐ Yes

☐ No

Do you know why you have been operated?

....

☐ I do not know

4.04. **In the past 2 weeks**, have you used any medicine for thyroid problems that were prescribed to you by a doctor?

☐ Yes

☐ No → **GO TO question 4.08**

4.05. Has the recipe been prescribed by a doctor from Luxembourg?

- ☐ Yes
- ☐ No

4.06. Do you know why you are taking medication for your thyroid?

....

- ☐ I do not know

4.07. Since how many years are you under medication?

☐☐ years

4.08. Do you regularly consume iodized salt?

- ☐ Yes, always
- ☐ Yes, from time to time
- ☐ No, never
- ☐ I do not know

5. RESPIRATORY HEALTH

Wheezing/Whistling

5.01. **In the last 12 months**, have you had any wheezing or whistling in the chest when breathing?

- ☐ Yes
- ☐ No → GO TO question 5.05 (Breathlessness)

5.02. **In the last 12 months**, have you had any wheezing or whistling when you did not have a cold?

- ☐ Yes
- ☐ No

5.03. **In the last 12 months**, how often on average has your sleep been disturbed due to wheezing or whistling in your chest?

- ☐ Never
- ☐ Awake less than 1 night in a week
- ☐ Awake 1 night or more in a week

5.04. Over **the last 12 months**, how much did wheezing or whistling in your chest interfere with your daily activities?

- ☐ Not at all
- ☐ A little bit
- ☐ Quite a bit
- ☐ A lot

Breathlessness

5.05. **In the last 12 months**, did you suffer from shortness of breath, breathlessness or breath difficulty, **except** in case of intense physical exercise?

- ☐ Yes
- ☐ No

5.06. Have you **ever** been bothered with shortness of breath when you go fast on level ground or going up a slight incline?

- ☐ Yes
- ☐ No → **GO TO question 5.11 (Cough)**
- ☐ Never walk on level ground or going up a slight incline
- ☐ Cannot walk → **GO TO question 5.11 (Cough)**

5.07. Are you short of breath, when you are walking with other people of similar age on level ground?

- ☐ Yes
- ☐ No
- ☐ Never walk with people of my own age on the flat foot

5.08. Do you need to stop and breathe when going at your own pace on level ground?

- ☐ Yes
- ☐ No

If 5.07 and 5.08 = NO, GO TO question 5.11 (Cough)

5.09. Do you need to stop and breathe when walking about 100 meters (or after a few minutes) on level ground?

- ☐ Yes
- ☐ No → **GO TO question 5.11 (Cough)**

5.10. Is your breathing so hard that you cannot leave the house or do you feel shortness of breath when dressing and undressing?

- ☐ Yes
- ☐ No

Cough

5.11. Do you have to cough, when you get up in the morning?

- ☐ Yes
- ☐ No

5.12. Do you usually cough during the day or night? (i.e. at least 6 fits of coughing per 24 hours)?

- ☐ Yes
- ☐ No

If 5.11 and 5.12 = NO, GO TO question 5.14 (Mucosities)

5.13. In a year, how many months in a row do you mostly cough this way?

- ☐ Less than 3 months
- ☐ 3 months
- ☐ More than 3 months

Mucosities

5.14. Do you usually have to coughing up glair in the morning after waking up?

- ☐ Yes
- ☐ No

5.15. Do you usually have to coughing up glair during the day or at night?

- ☐ Yes
- ☐ No

If question 5.14 and 5.15 = NO, GO TO question 5.17 (Respiratory symptoms)

5.16. In a year, how many months in a row do you regularly cough up glair?

- ☐ Less than 3 months
- ☐ 3 months
- ☐ More than 3 months

Respiratory symptoms

5.17. Did you suffer from an acute worsening of your respiratory symptoms **in the last 12 months** (wheezing/whistling, breathlessness, cough and phlegm)?

- ☐ Yes
- ☐ No → **GO TO question 5.20**

5.18. **In the last 12 months**, how often did you need, a treatment for acute respiratory worsening of your symptoms?

☐☐ times

- ☐ No treatment needed → **GO TO question 5.20**

5.19. How were these episodes of acute worsening of your respiratory symptoms usually treated?

- ☐ Antibiotics
- ☐ Corticosteroids / cortisone derivatives
- ☐ Inhalers
- ☐ Other
- ☐ I do not remember

If possible, specify the names of the medications:

....

....

....

....

Respiratory disease diagnosis

5.20. **During your life**, has ever one of the following diseases or health problems been diagnosed by a medical doctor?

Allergy: rhinitis, hay fever, eye inflammation, skin inflammation, food allergy or other allergy (except for allergic asthma)

☐ Yes

☐ No

Eye inflammation due to allergy	☐ Yes	☐ No
Nasal allergies including hay fever	☐ Yes	☐ No
Eczema or any kind of skin allergy	☐ Yes	☐ No
Food allergy	☐ Yes	☐ No
Asthma (allergic asthma included)	☐ Yes	☐ No
Emphysema	☐ Yes	☐ No
Chronic Bronchitis	☐ Yes	☐ No
COPD	☐ Yes	☐ No
Sleep apnoea	☐ Yes	☐ No

5.21. **In the past 12 months**, have you had any of the following diseases?

Allergy: rhinitis, hay fever, eye inflammation, skin inflammation, food allergy or other allergy (except for allergic asthma)

☐ Yes

☐ No

Eye inflammation due to allergy	☐ Yes	☐ No
Nasal allergies including Hay fever	☐ Yes	☐ No
Eczema or any kind of skin allergy	☐ Yes	☐ No
Food allergy	☐ Yes	☐ No
Asthma (allergic asthma included)	☐ Yes	☐ No
Emphysema	☐ Yes	☐ No
Chronic Bronchitis	☐ Yes	☐ No
COPD	☐ Yes	☐ No
Sleep apnoea	☐ Yes	☐ No

→ **If question 5.20 or 5.21 Asthma = YES, GO TO question 5.22**

→ **If question 5.20 ou 5.21 Emphysema = YES or Chronic Bronchitis = YES or COPD = YES, GO TO question 5.29**

→ **If question 5.20 and 5.21 = NO, GO TO page 108 (Medical examination)**

→ **If question 5.21 = NO, GO TO page 108 (Medical examination)**

ONLY FOR ASTHMA

5.22. How old were you when a doctor told you for the first time that you have asthma?

☐☐ years

5.23. How often have you had, **over the last 4 weeks**, daytime asthma symptoms?

- ☐ Never
- ☐ 2 times or less per week
- ☐ More than 2 times per week

5.24. How often have you had, **over the last 4 weeks**, asthma symptoms at night?

- ☐ Never
- ☐ 2 times or less per week
- ☐ More than 2 times per week

5.25. **Over the last 4 weeks**, how often did you wake up at night because of asthma symptoms?

- ☐ Never
- ☐ 2 times or less per week
- ☐ More than 2 times per week

5.26. How often, **over the last 4 weeks**, have your normal activities been disrupted by asthma symptoms?

- ☐ Never
- ☐ 2 times or less per week
- ☐ More than 2 times per week

5.27. How often did you require, **over the last 4 weeks**, treatment to relieve your asthma symptoms or did you need a treatment in emergency case / asthma attack?

- ☐ Never
- ☐ 2 times or less per week
- ☐ More than 2 times per week

5.28. When did you have your last asthma attack?

- ☐ Less than 4 weeks ago
- ☐ More than 4 weeks but less than 12 months
- ☐ Before 1 year to 5 years
- ☐ Before more than 5 years

ONLY FOR EMPHYSEMA / CHRONIC BRONCHITIS / COPD

5.29. How old were you when a doctor has told you for the first time that you have emphysema or chronic bronchitis or COPD (chronic obstructive pulmonary disease)?

☐☐ years

5.30. **Over the last 12 months**, did you have any exacerbations / flare ups (when symptoms were worse than usual for the least two days in a row)?

- ☐ Yes
- ☐ No → **If NO, GO TO question 5.34 (Medication)**

5.31. How many exacerbations / flare ups did you have **in the last 12 months**?

- ☐ One
- ☐ Two
- ☐ Three or more

5.32. How many times, **over the last 12 months**, have you been admitted to hospital for at least one night because of an exacerbation / flare up?

☐☐☐ times

5.33. How many times, **over the last 12 months**, did you go to the emergency room because of an exacerbation / flare up?

☐☐☐ times

Medication

5.34. **In the past 2 weeks**, did you take any medicine prescribed for you by a doctor against the following diseases?

Asthma (allergic asthma included)	☐ Yes	☐ No
Allergic symptomes excluding asthma (eczema, rhinits, hay fever)	☐ Yes	☐ No
Chronic Bronchitis	☐ Yes	☐ No
COPD	☐ Yes	☐ No
Emphysema	☐ Yes	☐ No

5.35. Have you **within the last 2 weeks** taken any medicine not prescribed for you by a doctor against allergy symptoms (eczema, rhinitis, hay fever)?

- ☐ Yes
- ☐ No

5.36. **In the past 12 months**, did you take any medicine prescribed for you by a doctor against the following diseases?

Asthma (allergic asthma included)	☐ Yes	☐ No
Allergic symptomes excluding asthma (eczema, rhinits, hay fever)	☐ Yes	☐ No
Chronic Bronchitis	☐ Yes	☐ No
COPD	☐ Yes	☐ No
Emphysema	☐ Yes	☐ No

5.37. **In the past 12 months**, have you used any medicines not prescribed by a doctor for allergic symptoms (eczema, rhinitis, hay fever)?

☐ Yes

☐ No

5.38. Are you currently in treatment for sleep apnea?

☐ Yes

☐ No → **GO TO next page: Medical Examination**

5.39. How your sleep apnea is usually treated?

☐ CPAP – Continous Positive Airway Pressure

☐ BiPAP – Biphasic Positive Airway Pressure

☐ LTOT – Long Term Oxygen Therapy

☐ Other: ....

MEDICAL EXAMINATION

1. BLOOD PRESSURE

1.01. Room temperature

☐☐ , ☐ °C

1.02. Has the participant done any of the following activities **1 hour before examination?**

Strenuous physical activity	☐ Yes	☐ No
Smoke	☐ Yes	☐ No
Eat something	☐ Yes	☐ No
Drink something other than water	☐ Yes	☐ No
Go to toilet	☐ Yes	☐ No

1.03. Measurement of blood pressure **on the right arm**

Arm used: ☐ **right (priority!)** ☐ left

Before the first measurement, the participant must remain ***seated*** *for at least* ***5 minutes, then 1 minute between each measurement.***

	1st	2nd	3rd
Systolic pressure (mmHg):	☐☐☐	☐☐☐	☐☐☐
Diastolic pressure (mmHg):	☐☐☐	☐☐☐	☐☐☐
No measurement:	☐	☐	☐
Error code of device:	....	....	....
Pulse (/60 sec):	☐☐☐	☐☐☐	☐☐☐

1.04. Type of device

☐ OMRON MX3 Plus

☐ OMRON M6 Comfort

Identification number of device: ☐

1.05. Selection of cuff

Arm measurement: ☐☐ , ☐ cm **(obligatory !)**

Cuff used:

☐ Small-Medium for OMRON MX3 Plus (22-32 cm)

☐ Large for OMRON MX3 Plus (32-42 cm)

☐ Small-Medium-Large for OMRON M6 Comfort (22-42 cm)

1.06. **If you used the left arm**, give the reason:

☐ Right arm paralyzed or spastic

☐ Amputation of the right arm

☐ Right arm in plaster

☐ Eczema on the right arm

☐ Shunt on the right arm

☐ Malformation of the right arm which hinders to fix the cuff

☐ Problems with lymphatic glands on the right arm, e.g. after mastectomy after breast cancer which hinders to fix the cuff

☐ Other: ....

1.07. Position of the participant during blood pressure measurement

☐ Sitting

☐ Lying down

1.08. **If the participant has been lying**, give the reason:

- ☐ Bedridden
- ☐ Other: ....

1.09. Reason why the blood pressure could not be measured

- ☐ Amputation of both arms
- ☐ Plaster on both arms
- ☐ Open sore on both arms
- ☐ Crucial on both arms
- ☐ Malformation on both arms which hinders to fix the cuff
- ☐ Problems with the lymphatic glands, e.g. after mastectomy on both sides after breast cancer which hinders to fix the cuff
- ☐ Refusal
- ☐ Other: ....

1.10. Observations:

....

....

....

....

....

....

....

2. ANTHROPOMETRY

2.1. Height

2.1.1. Measurement of the height

☐☐☐ , ☐ cm

☐ Not measured

2.1.2. Identification number of device

☐

2.1.3. Reason why the size has not been measured

☐ Wheelchair or immobile

☐ Unsteady when standing

☐ Height bigger than the limit of the stadiometer

Specify the upper limit of the stadiometer: ☐☐☐ , ☐ cm

☐ Haircut/hairstyle or hat/turban which hinders the measurement (impossible to remove)

☐ Refusal

☐ Other: ....

2.1.4. Observations:

....

....

....

....

....

....

2.2. Weight

2.2.1. Measurement of the weight

[ ][ ][ ] , [ ][ ] Kg

☐ Not measured

2.2.2. Identification number of device

[ ]

2.2.3. The measurement has been made

☐ **In underwear (priority!)**

☐ With light clothing, specify: ....

☐ Other: ....

2.2.4. Reason why the weight has not been measured

☐ Wheelchair or immobile

☐ Unsteady when standing

☐ Weight bigger than the limit of the scale

Specify the upper limit of the scale: [ ][ ][ ] , [ ][ ] Kg

☐ Refusal

☐ Other: ....

2.2.5. Observations:

....

....

....

....

....

....

2.3. Waist size

2.3.1. Waist size

☐☐☐ , ☐ cm

☐ Not measured

2.3.2. Identification number of the tape measure

☐

2.3.3. The measurement has been made

☐ **Directly on the skin (priority!)**

☐ In underwear

☐ On light clothing, specify: ....

☐ Other: ....

2.3.4. Reason why the waist size has not been measured

☐ Wheelchair, immobile or cannot stand

☐ Unsteady when standing

☐ Waist size larger than the tape measure

Maximum length of the tape measure: ☐☐☐ , ☐ cm

☐ Significant hernia, stoma or other disturbing things on the measuring zone:

....

☐ Refusal

☐ Other: ....

2.3.5. Observations:

....

....

....

....

2.4. Hip size

2.4.1. Hip size

[ ][ ][ ] , [ ] cm

☐ Not measured

2.4.2. The measurement has been made

☐ **In underwear (priority!)**

☐ With light clothing, specify: ....

☐ Other: ....

2.4.3. Reason why the hip size has not been measured

☐ Wheelchair, immobile or cannot stand

☐ Unsteady when standing

☐ Device or anything placed on the measuring zone

☐ Refusal

☐ Other: ....

2.4.4. Observations:

....

....

....

....

....

....

....

....

2.5. Thigh size

2.5.1. **Right (priority!)** thigh size

Proximal: ☐☐☐ , ☐ cm

☐ Not measured

→ **If NO**, give the reason: ....

The proximal thigh measurement has been made

☐ **Directly on the skin (priority!)**

☐ With light clothing, specify: ....

☐ Other: ....

Mid-thigh: ☐☐☐ , ☐ cm

☐ Not measured

→ **If NO**, give the reason: ....

The mid-thigh measurement has been made

☐ **Directly on the skin (priority!)**

☐ With light clothing, specify: ....

☐ Other: ....

2.5.2. Which side has been measured?

- ☐ **Right (priority!)**
- ☐ Left

→ **If you mesured left thigh,** give the reason

- ☐ Right leg paralyzed or spastic
- ☐ Amputation of the right leg
- ☐ Right leg in plaster
- ☐ Eczema on right leg
- ☐ Intravenous device placed on the right leg
- ☐ Malformation of the right leg which hinders the measurement
- ☐ Other: ....

2.5.3. Reason why the **proximal leg size** has not been measured

- ☐ Wheelchair, immobile or cannot stand
- ☐ Very wobbly when standing
- ☐ Device or anything placed on the measuring zone
- ☐ Refusal
- ☐ Other: ....

2.5.4. Reason why the **mid-thigh leg size** has not been measured

- ☐ Wheelchair, immobile or cannot stand
- ☐ Very wobbly when standing
- ☐ Device or anything placed on the measuring zone
- ☐ Refusal
- ☐ Other: ....

2.5.5. Observations:

....

....

3. ECG

3.01. Are you followed by a cardiologist?

- ☐ Yes
- ☐ No

3.02. Do you have one of the following diseases?

Congenital heart defect	☐ Yes	☐ No	☐ Do not know
Arrhytmia	☐ Yes	☐ No	☐ Do not know
Mitral regurgitation (valvular)	☐ Yes	☐ No	☐ Do not know

3.03. Have you have **ever** carried out any of the following measures in your whole life?

Coronarography	☐ Yes	☐ No	☐ Do not know
Reanimation / cardiac arrest	☐ Yes	☐ No	☐ Do not know
Bypass	☐ Yes	☐ No	☐ Do not know
Angioplasty	☐ Yes	☐ No	☐ Do not know
Valve prostheses	☐ Yes	☐ No	☐ Do not know
Pacemaker	☐ Yes	☐ No	☐ Do not know
Defibrillator intern	☐ Yes	☐ No	☐ Do not know
Heart transplantation	☐ Yes	☐ No	☐ Do not know

3.04. **Did your father, during his life**, have one of the following diseases?

Myocardial infarction	☐ Yes	☐ No	☐ Do not know
**If YES**, at what age? ☐☐ years<br>☐ Do not know			
Congenital heart failure	☐ Yes	☐ No	☐ Do not know
Arrhythmia	☐ Yes	☐ No	☐ Do not know
Mitral insufficiency	☐ Yes	☐ No	☐ Do not know
High blood pressure	☐ Yes	☐ No	☐ Do not know
Diabetes	☐ Yes	☐ No	☐ Do not know
**If YES**, at what age? ☐☐ years<br>☐ Do not know			
Hypercholesterolemia	☐ Yes	☐ No	☐ Do not know
Stroke	☐ Yes	☐ No	☐ Do not know
**If YES**, at what age? ☐☐ years<br>☐ Do not know			

3.05. **Did your mother, during her life**, have one of the following diseases?

Myocardial infarction	☐ Yes	☐ No	☐ Do not know
**If YES**, at what age? ☐☐ years<br>☐ Do not know			
Congenital heart failure	☐ Yes	☐ No	☐ Do not know
Arrhythmia	☐ Yes	☐ No	☐ Do not know
Mitral insufficiency	☐ Yes	☐ No	☐ Do not know
High blood pressure	☐ Yes	☐ No	☐ Do not know
Diabetes	☐ Yes	☐ No	☐ Do not know
**If YES**, at what age? ☐☐ years<br>☐ Do not know			
Hypercholesterolemia	☐ Yes	☐ No	☐ Do not know
Stroke	☐ Yes	☐ No	☐ Do not know
**If YES**, at what age? ☐☐ years<br>☐ Do not know			

3.06. **Did your brothers/sisters, in their lifetime**, have one of these following diseases?

Only child ☐ Yes ☐ No

Myocardial infarction	☐ Yes	☐ No	☐ Do not know
Congenital heart failure	☐ Yes	☐ No	☐ Do not know
Arrhythmia	☐ Yes	☐ No	☐ Do not know
Mitral insufficiency	☐ Yes	☐ No	☐ Do not know
High blood pressure	☐ Yes	☐ No	☐ Do not know
Diabetes	☐ Yes	☐ No	☐ Do not know
Hypercholesterolemia	☐ Yes	☐ No	☐ Do not know
Stroke	☐ Yes	☐ No	☐ Do not know

3.07. Medical diagnosis

The interpretation of the ECG will be done by a cardiologist!

☐ ECG unremarkable

☐ ECG with anomaly, and suggestion that participant consults his/her family physician or a cardiologist

3.08. Observations of the cardiologist

....

....

....

....

....

....

3.09. Identification number of ECG device

☐

3.10. ECG has been made

☐ Yes

☐ No

If NO, give the reason:

☐ Refusal

☐ Other: ....

If the participant has a pacemaker, did you use a magnet in the implementation of the ECG?

☐ Yes

☐ No

☐ **NOT APPLICABLE**

If NO, give the reason:

☐ Refusal

☐ Anxiety/Fear

☐ Other: ....

3.11. Observations:

....

....

....

....

....

....

4. SPIROMETRY

Before beginning of the test, you must check if the participant does not have any of the following contraindications below.

4.01. Reason(s) why the spirometry test cannot be done:

- ☐ The participant is pregnant
- ☐ Abdominal or thoracic surgery during the last 3 months
- ☐ Myocardial infarction during the last 3 months
- ☐ Stay in the hospital for heart complaints in the last month
- ☐ Pneumothorax in the last 3 months
- ☐ Retinal or eye surgery during the last 3 months
- ☐ Operation of the ears in the last 3 months
- ☐ Treated for tuberculosis
- ☐ Acute respiratory diseases
- ☐ Resting pulse over 120 beats/min
- ☐ Refusal
- ☐ Other: ....
- ☐ NONE OF THE REASONS MENTIONED ABOVE

4.02. Identification number of the spirometer

☐

Respect the procedure while doing the test.

4.03. The spirometry test has been performed

- ☐ Yes
- ☐ No

4.04. The participant has used a bronchodilatator before the test (the study nurse was also there)?

☐ Yes

☐ No

4.05. Number of exhales measured (max 8)

☐

4.06. Number of exhales considered as acceptable (ideally 3)

☐

4.07. Measures in conformity with the criteria ATS/ERS

☐ Yes

☐ No

4.08. Observations:

....

....

....

....

....

....

....

....

....

5. VISUAL ACUITY

5.01. Does the participant wear glasses or contact lenses?

- ☐ Yes
- ☐ No

If YES, what kind of vision problems does he/she suffer from?

- ☐ Myopia (nearsighted/shortsighted)
- ☐ Hypermetropia (longsightedness)
- ☐ Astigmatism (vision is blurred)
- ☐ Presbyopia (condition where with age, the eye exhibits a progressively diminished ability to focus on near objects)
- ☐ Other: ....

The participant MUST wear his/her glasses or contact lenses!

5.02. Did the participant bring his glasses or contact lenses?

- ☐ Yes
- ☐ No
- ☐ Not wearing glasses/contact lenses

5.03. Brightness

☐☐☐☐ Lux

5.04. Raskin scale with **the big E**: Near vision (33 cm), write down the **last line** that has been ridden.

LEFT EYE	RIGHT EYE
☐ **/8 lines**	☐ **/8 lines**
☐ Not measured	☐ Not measured
☐ Not applicable	☐ Not applicable

5.05. Snellen scale with **multiple letters (people who have literacy)**: Distance vision (5 m), write down the **last line** that has been ridden.

LEFT EYE

☐ **/9 lines**

☐ Not measured

☐ Not applicable

RIGHT EYE

☐ **/9 lines**

☐ Not measured

☐ Not applicable

5.06. Raskin scale with **the big E (people who DO NOT have literacy)**: Distance vision (5 m), write down the **last line** that has been ridden.

LEFT EYE

☐ **/10 lines**

☐ Not measured

☐ Not applicable

RIGHT EYE

☐ **/10 lines**

☐ Not measured

☐ Not applicablee

5.07. Reason why the visual acuity has not been measured

☐ Participant is blind

☐ Refusal

☐ Lost of the left eye

☐ Lost of the right eye

☐ Other: ....

5.08. Observations:

....

....

....

....

....

6. HAIR

6.01. Hair sample has been taken:

☐ Yes

☐ No

If NO, give the reason:

☐ No hair or shaved

☐ Refusal

☐ Other: ....

6.02. **Natural** hair color:

*If the person has white or gray hair, write **white** or **gray, NOT** the original color.*

☐ White

☐ Gray

☐ Light blond

☐ Dark blond

☐ Red

☐ Auburn

☐ Ash brown

☐ Light brown

☐ Dark brown

☐ Black

6.03. Has the participant treated hair (permed, dyed hair, etc.)?

- ☐ Yes
- ☐ No

If YES, please specify:

More than one answer possible.

- ☐ Coloured/dyed
- ☐ Bleaching
- ☐ Perm
- ☐ Other: ....

6.04. Did the participant apply products on his hair?

- ☐ Yes
- ☐ No

If YES, please specify:

More than one answer possible.

- ☐ Cosmetics: gel, hair spray, etc.
- ☐ Drugs therapy, specify:

....

End of the Study

Time end of the 2nd part

__ __ : __ __

HH:MM
